# Probe Region Expression Estimation for RNA-Seq Data for Improved Microarray Comparability

**DOI:** 10.1371/journal.pone.0126545

**Published:** 2015-05-12

**Authors:** Karolis Uziela, Antti Honkela

**Affiliations:** 1 Helsinki Institute for Information Technology HIIT, Department of Computer Science, University of Helsinki, Helsinki, Finland; 2 Department of Biochemistry and Biophysics, Science for Life Laboratory, Stockholm University, 17121 Solna, Sweden; Indiana University Bloomington, UNITED STATES

## Abstract

Rapidly growing public gene expression databases contain a wealth of data for building an unprecedentedly detailed picture of human biology and disease. This data comes from many diverse measurement platforms that make integrating it all difficult. Although RNA-sequencing (RNA-seq) is attracting the most attention, at present, the rate of new microarray studies submitted to public databases far exceeds the rate of new RNA-seq studies. There is clearly a need for methods that make it easier to combine data from different technologies. In this paper, we propose a new method for processing RNA-seq data that yields gene expression estimates that are much more similar to corresponding estimates from microarray data, hence greatly improving cross-platform comparability. The method we call PREBS is based on estimating the expression from RNA-seq reads overlapping the microarray probe regions, and processing these estimates with standard microarray summarisation algorithms. Using paired microarray and RNA-seq samples from TCGA LAML data set we show that PREBS expression estimates derived from RNA-seq are more similar to microarray-based expression estimates than those from other RNA-seq processing methods. In an experiment to retrieve paired microarray samples from a database using an RNA-seq query sample, gene signatures defined based on PREBS expression estimates were found to be much more accurate than those from other methods. PREBS also allows new ways of using RNA-seq data, such as expression estimation for microarray probe sets. An implementation of the proposed method is available in the Bioconductor package “prebs.”

## Introduction

Public gene expression databases such as ArrayExpress [1] and Gene Expression Omnibus [2] host public data from more than half a million gene expression experiments. While the field is moving toward sequencing-based methods for expression analysis, an overwhelming majority of the existing and even newly uploaded data in these databases are still from microarray platforms as demonstrated in Table 1. The existing microarray-based data represent a huge investment and being able to utilise it efficiently as background information in new sequencing-based studies is of great interest.

**Table 1:** Number of RNA-seq and microarray experiments in ArrayExpress and GEO databases.

**Platform**	**Database**	**2010**	**2011**	**2012**	**2013**	**2014**	**2010–2014**	**All time**
**RNA-seq**	**ArrayExpress**	269	499	877	1454	2114	**5213**	**5470**
**RNA-seq**	**GEO**	136	309	567	1038	1741	**3791**	**3976**
**Microarray**	**ArrayExpress**	6032	5604	6052	6528	5822	**30038**	**40525**
**Microarray**	**GEO**	4243	5152	5521	5705	5589	**26210**	**42130**

The data are valid as of January 25, 2015. When querying ArrayExpress database, option “ArrayExpress” data only was unchecked.

Recently there has been significant interest in utilising the large public databases to holistically characterise phenotypes based on expression in new samples [3]. Most work utilising these large databases is based on differential expression [4–6], but Schmid *et al.* [3] argue that absolute expression can yield a more comprehensive picture. All of these methods are currently restricted to microarray data, which severely limits their utility in new studies.

RNA-seq and microarrays are based on very different principles and ultimately measure different things [7]. Numerous experimental comparisons have demonstrated RNA-seq and microarrays to yield broadly comparable results [8–15]. These results demonstrate that the platforms typically agree on differentially expressed genes between sufficiently different samples, although RNA-seq tends to be more sensitive. For measures of absolute expression, there is typically a clear correlation, the level of which ranges from moderate to very high depending on the example.

In this paper we present a method for processing RNA-seq data in a way to make the resulting expression measures significantly more comparable with measures derived from microarray data by estimating the expression level at the microarray probe regions using a method we call PREBS (Probe Region Expression estimation Based on Sequencing). The improvement is especially significant for measures of absolute expression. This improved comparability comes at the expense of ignoring some information in the RNA-seq data by focusing the analysis to regions covered by the microarray probes. Because of this loss of information, PREBS should not be viewed as a replacement of standard RNA-seq analysis tools. Neither is it a replacement for actually performing the corresponding microarray experiment if the sample material and sufficient resources are available, but rather a cheap computational alternative for the very common case when either samples or resources are not available.

## Materials and Methods

### Basic description of the method

One of the fundamental differences between microarray and RNA-seq technologies is that microarrays, especially now ubiquitous oligonucleotide arrays, measure gene expression based on the parts of the gene where probe sequences are located [16] while RNA-seq measures expression over the whole gene sequence [17]. The idea of our method is to eliminate this difference by calculating RNA-seq gene expression measures only based on the parts of the gene where microarray probe sequences are located.

Traditionally gene expression is estimated from RNA-seq data by counting the number of reads that overlap with exons of the gene (count methods) [17, 18]. The analysis in higher eukaryotes can be complicated by alternative splicing. To account for this, several methods have been proposed that are based on deconvolution of transcript isoform expression using probabilistic models [19–22], but these methods still estimate the expression level across the whole gene.

In PREBS method we estimate probe region expression by counting the number of reads that overlap with probe regions and using a statistical model to infer the expression level from the read counts. We treat the inferred probe region expression levels in a similar way as they are treated in computational microarray processing pipelines. In particular, we apply two different types of microarray data summarisation algorithms used for Affymetrix data analysis: the classical RMA algorithm [23] and as an example of more modern probabilistic methods also the RPA algorithm [24]. The details of applying summarisation algorithms and the statistical model used to infer probe regions will be described in later sections.

Using the described method we aim to computationally process RNA-seq data in a way that is similar to microarray computational processing pipelines. In the Results section we show that gene expression measures that we get from RNA-seq data this way are more similar to microarray measures than the measurements that we get using conventional RNA-seq data processing methods. We call our RNA-seq data processing method PREBS (Probe Region Expression estimation Based on Sequencing).

### Read counting

For counting read overlaps PREBS uses count_overlaps() function from GenomicRanges package in R/Bioconductor. Just like implemented in count_overlaps() function, PREBS counts the read for all overlapping probe regions, even if one read overlaps with several of them. There is no need to discard reads that overlap several probe regions, because it would cause biased under-expression of densely probe-packed genome areas. Moreover, PREBS has inherited a feature from count_overlaps() function that allows to select whether the strand from which the read originates should be ignored when counting the overlaps. Since most of the RNA-seq protocols that are used nowadays are not strand-specific, the default behaviour of PREBS is to ignore the strand. Finally, PREBS supports a possibility to process both single-ended and paired-ended reads. If paired-ended mode is selected, the two mates are treated as a single unit, not as independent reads during read-counting process.

### Probe region expression estimation from RNA-seq

Read sampling in sequencing is inherently a stochastic process. To account for the uncertainty this induces, we use statistical methods to infer the probe region expression level from read data.

We assume that the number of reads from a region with a given expression level follows the Poisson distribution. Placing a conjugate gamma prior on the expression level, we obtain an estimate of the expression level as the mean of the posterior distribution. The hyperparameters of the prior are determined using an empirical Bayesian approach by maximising the marginal likelihood of the full data.

### Expression summarisation

Affymetrix microarray probes are grouped into probe sets containing 8–20 perfect match / mismatch probe pairs. Perfect match probes are completely complementary to gene portion they are interrogating while mismatch probes have their middle nucleotide changed. Some algorithms like MAS5 [25] use expression values from mismatch probes to account for non-specific binding while RMA and RPA completely ignore mismatch probe values and use only the perfect match probes.

RMA is probably the most popular microarray summarisation algorithm used nowadays. RMA models probe-specific affinities [23], but it does not model probe-specific variances that are modelled by newer summarisation algorithms such as RPA [24]. We have implemented these two summarisation modes in the PREBS algorithm: RMA and RPA. The user has a possibility to choose one of these two summarisation algorithms when running PREBS.

Our implementation of PREBS uses the original RMA and RPA code from Affy [26] and RPA [24] packages respectively and applies them on our probe expression estimates. The noise characteristics of microarrays and RNA-seq are different, especially at the low end of the expression level spectrum, where microarrays have a significant background that is removed by the background correction step in the RMA and RPA algorithms. Because of the digital nature of RNA-seq, there is no explicit background like in microarrays, and hence the same background correction is not applicable. Low expression values in RNA-seq are less accurate and can be considered as a background, but they can be effectively dealt with by filtering as indicated below. Therefore, when we applied RMA and RPA algorithms on our data, we have skipped the background correction step. The two other major steps of RMA and RPA algorithms, normalisation and summarisation, were left unchanged and performed as they are implemented in corresponding packages.

When processing microarray data using RMA or RPA algorithm, the user has two options: process the data based on original microarray probe set definitions or based on alternative probe set definitions using so called Custom CDF files [27]. By default, the resulting expression values are calculated for the original microarray probe sets. On the other hand, when the data are processed using Custom CDF files, the expression measures can be directly calculated for other units such as Ensembl genes. The latter option greatly simplifies the comparison between microarray and RNA-seq data, since microarray gene expression values calculated for Ensembl genes can be directly compared with the gene expression values calculated using various RNA-seq data processing tools.

PREBS shares the feature of being able to run in the same two modes. On the one hand, the values that we get using Custom CDF files for Ensembl genes can be easily compared with RNA-seq gene expression values and therefore, most of the results in this paper are based on this mode. On the other hand, being able to get expression values for the original probe sets is a unique feature of PREBS that no other RNA-seq data processing method possesses. This feature is certainly very useful for people who prefer to work on expression summaries for microarray probe sets but still want to compare these to RNA-seq expression estimates.

### Tools used for implementation

In order to evaluate the effectiveness of our method (PREBS) we compared it to representatives of two RNA-seq analysis methods: count-based [17] (“read counting”) and isoform deconvolution (“MMSEQ”). We processed sequencing data using each of the methods and evaluated their agreement with microarray data by calculating correlations of gene expression.

For the PREBS method, reads were mapped by TopHat software version 1.4.1 [28] to Human genome version GRCh37.65. We considered only unique genomic alignments to annotated transcripts. When running PREBS with Ensembl gene summaries, the locations for probe regions were retrieved from Custom CDF file annotations (version 15.0.0 ENSG) [27]. For probe set summaries, we mapped the probe sequences to the latest human genome build (hg19) using Bowtie (version 0.12.7). The read overlaps with probe regions were calculated using GenomicRanges package from R/Bioconductor [29]. Probe region expression estimates were calculated as described above and fed to the RMA and RPA algorithms from R/Bioconductor Affy (version 1.42.2) and RPA (version 1.20.01) packages, respectively.

Read counting RPKM values were calculated using the same tools as in PREBS method, but read overlap counts were calculated for Ensembl genomic annotations that were downloaded using GenomicFeatures package. RPKM values were calculated using these counts and log_2_ values were taken.

For isoform deconvolution we used MMSEQ [21] (software version 0.9.18). Bowtie software (version 0.12.7) [30] was used to map the reads to the transcriptome, as recommended by MMSEQ manual. MMSEQ options were set to default and Bowtie options were set as recommended by MMSEQ (-a –best –strata -S -m 100 -X 400). Human transcriptome version GRCh37.65 from Ensembl database was used. MMSEQ output values were converted from natural logarithm scale to log_2_ scale.

Microarray expression values were summarised using RMA and RPA algorithms. In case of multiple replicates, the mean value was taken as an absolute expression estimate for each state. RMA and RPA summarised values were in log_2_ scale, so no logarithm base conversion was needed.

Significance tests between the observed correlation differences were performed using r.test() function from psych package in R.

## Results

### Data sets

We evaluated the performance of our method on two data sets: Marioni [8] and Acute myeloid leukaemia (LAML) from The Cancer Genome Atlas (TCGA) database [31]. These will be referred to as Marioni and LAML data sets, respectively. Both of these data sets have paired RNA-seq and microarray data. The Marioni data set has two samples, human kidney and liver, both of which were used for testing. The LAML data set has 200 samples in all, 163 of which have both microarray and RNA-seq data available. For 16 of those read mapping using TopHat failed to complete (sample numbers: 2808, 2813, 2823, 2824, 2844, 2853, 2865, 2868, 2888, 2892, 2912, 2917, 2959, 2973, 2980, 2982) so we skipped those samples and used the remaining 147 samples. In both of the data sets RNA-seq platform is Illumina Genome Analyzer II and microarray platform is Affymetrix U133 Plus 2.

The main criterion for selecting the data sets was availability of both RNA-seq and microarray data for exactly the same samples that would be prepared in the same way. There are very few data sets meeting this criterion. In the other data sets that had paired RNA-seq–microarray data either the samples were different or not prepared in the same way, or they had some other technical problems (raw data not available, the pairing of the samples is not clear etc.).

We were interested in checking how much information is lost in each RNA-seq data set by only focusing on microarray probe locations in the PREBS method. For that we computed ratios of how many of the total reads were mapped to gene regions and out of those how many were mapped to the microarray probe locations. In the Marioni data set on average 79.4% of the reads where mapped to gene regions. Out of these, 21.1% where mapped to microarray probe locations inside the gene regions. In the LAML data set on average 59.1% reads were mapped to gene regions and 25.2% of these were mapped to probe locations.

### Absolute expression comparison

We ran PREBS both in RMA and RPA modes and compared it with two other RNA-seq data processing methods: count-based [17] (“read counting”) and isoform deconvolution (“MMSEQ”) [21]. PREBS and the other two RNA-seq data processing methods were evaluated based on agreement with microarray expressions where microarray data was processed using the same RMA and RPA methods. We found that two microarray data processing methods give very similar results. Overall, the RNA-seq data processing methods show a slightly higher agreement with microarrays when RPA method is used (see S10 Fig and S11 Fig). Therefore, in the main text we include only the plots where microarray data were processed with RPA method and PREBS was run in RPA mode while in the supplementary material we provide all of the corresponding plots where microarray data were processed with RMA method and PREBS was run in RMA mode.

First, we will present results for expression summaries for Ensembl genes both from microarray data and PREBS. This ensures a fair comparison against the other RNA-seq data processing methods, as the methods we tested are able to calculate expression values for Ensembl genes, too. The other two RNA-seq data processing methods that PREBS was compared to were count-based [17] (“read counting”) and isoform deconvolution (“MMSEQ”) [21].

In most gene expression studies, low expressed genes are filtered out, because their measurements are noisy and unreliable. Common filtering thresholds for RNA-seq data vary around 0.3 RPKM [32]. This fraction accounts for 70% of top expressed genes in the Marioni data set and 60.9% of top expressed genes in the LAML data set. To make the filtering uniform among all of the data sets and methods, we have decided to use at most 60% of top expressed genes.

**Fig 1:**
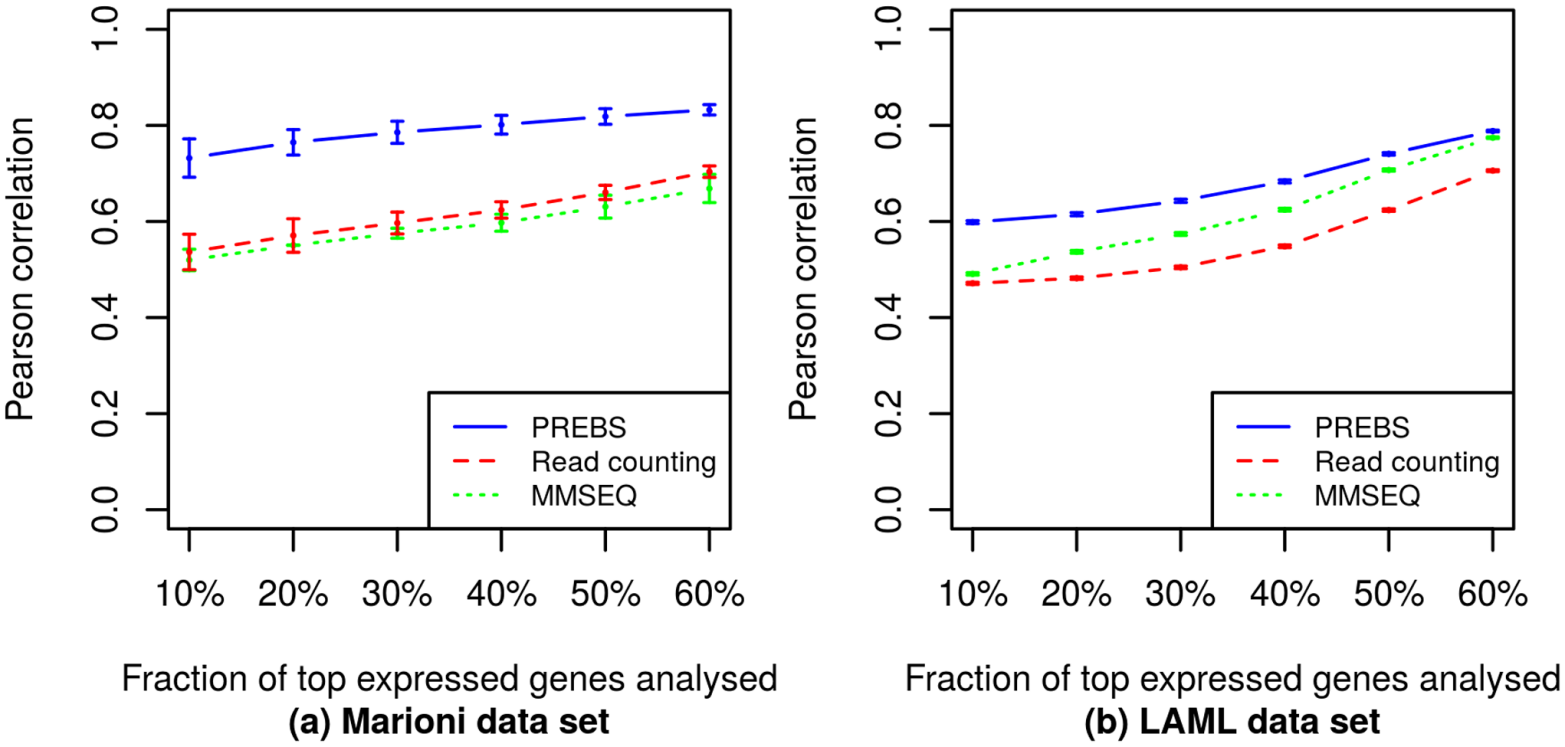
Averaged absolute gene expression correlations (RPA mode). The plots show average absolute gene expression correlations between different RNA-seq data processing methods and the microarray. Different points correspond to different numbers of top expressed genes. The correlations are averaged over all samples in the corresponding data sets: (a) the Marioni *et al.* data set, (b) the LAML data set. The error bars correspond to standard errors of the mean. For LAML data set the standard errors are so small that the top and bottom error bars are merged in the plot.

In order to evaluate the agreement of each RNA-seq data processing method (PREBS, MMSEQ and read counting) with microarrays, we have calculated the Pearson correlation of sequencing-based expression values with microarray expression values for each sample in Marioni and LAML data sets. The correlations were calculated for different fractions of most highly expressed genes in a sample: 10–60%. To evaluate the methods performance for whole data sets, we took an average correlation over all samples in each data set (2 samples in the Marioni data set and 147 samples in the LAML data set). We provide the resulting graph that shows the average Pearson correlations plotted as a function of the fraction of most highly expressed genes (Fig 1).

From Fig 1 we can clearly see that PREBS has the best agreement with microarrays for any number of top expressed genes taken in both data sets. The differences in the LAML data set are highly statistically significant (*p* < 10^−15^ for Wilcoxon signed-rank test) while the Marioni data set is too small to obtain statistically significant results. Moreover, we observe that the difference is larger for smaller fractions of top expressed genes taken. This suggests that PREBS is especially useful when focusing on highly expressed genes.

**Fig 2:**
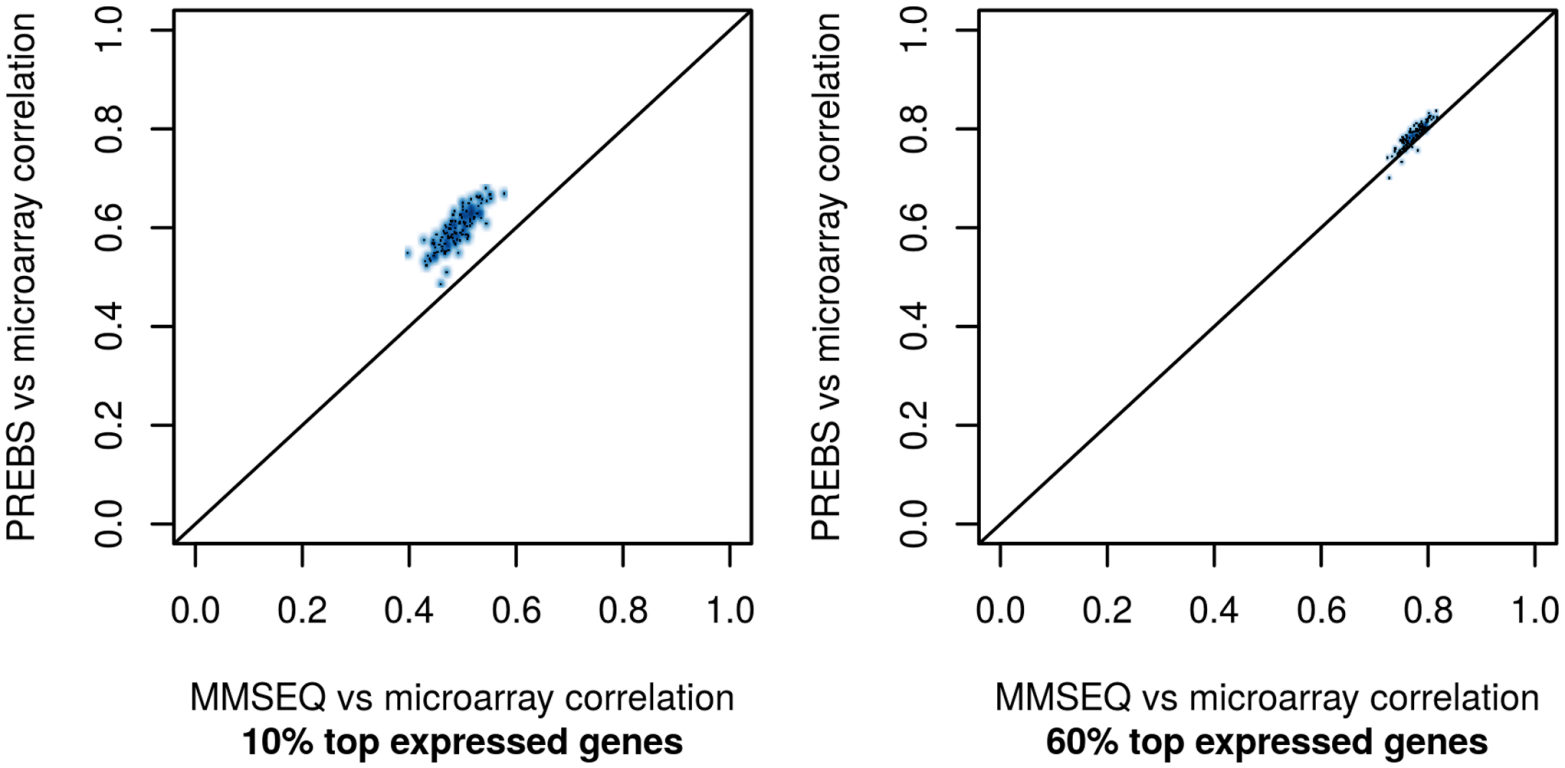
Absolute gene expression correlation scatter plots (RPA mode). The plots show the comparison of correlations of PREBS vs microarray and MMSEQ vs microarray for all of the samples in the LAML data set. Each point represents one sample. Two different percentages of top expressed genes are taken: (a) 10%, (b) 60%.

In order to show that the difference in correlations is robust among different samples, we provide correlation scatter plots (Fig 2). Each point in the plot represents the comparison of correlation between PREBS vs microarray and MMSEQ vs microarray for a single sample in the LAML data set (so there are 147 points in each of the plots). PREBS correlation with microarray is better than MMSEQ correlation with microarray for all of the points that are above the diagonal. From these plots we can see that PREBS agreement with microarray is consistently better than MMSEQ among different samples in the LAML data set. Moreover, we can see again that the difference in performance is larger when we take only 10% of top expressed genes.

**Fig 3:**
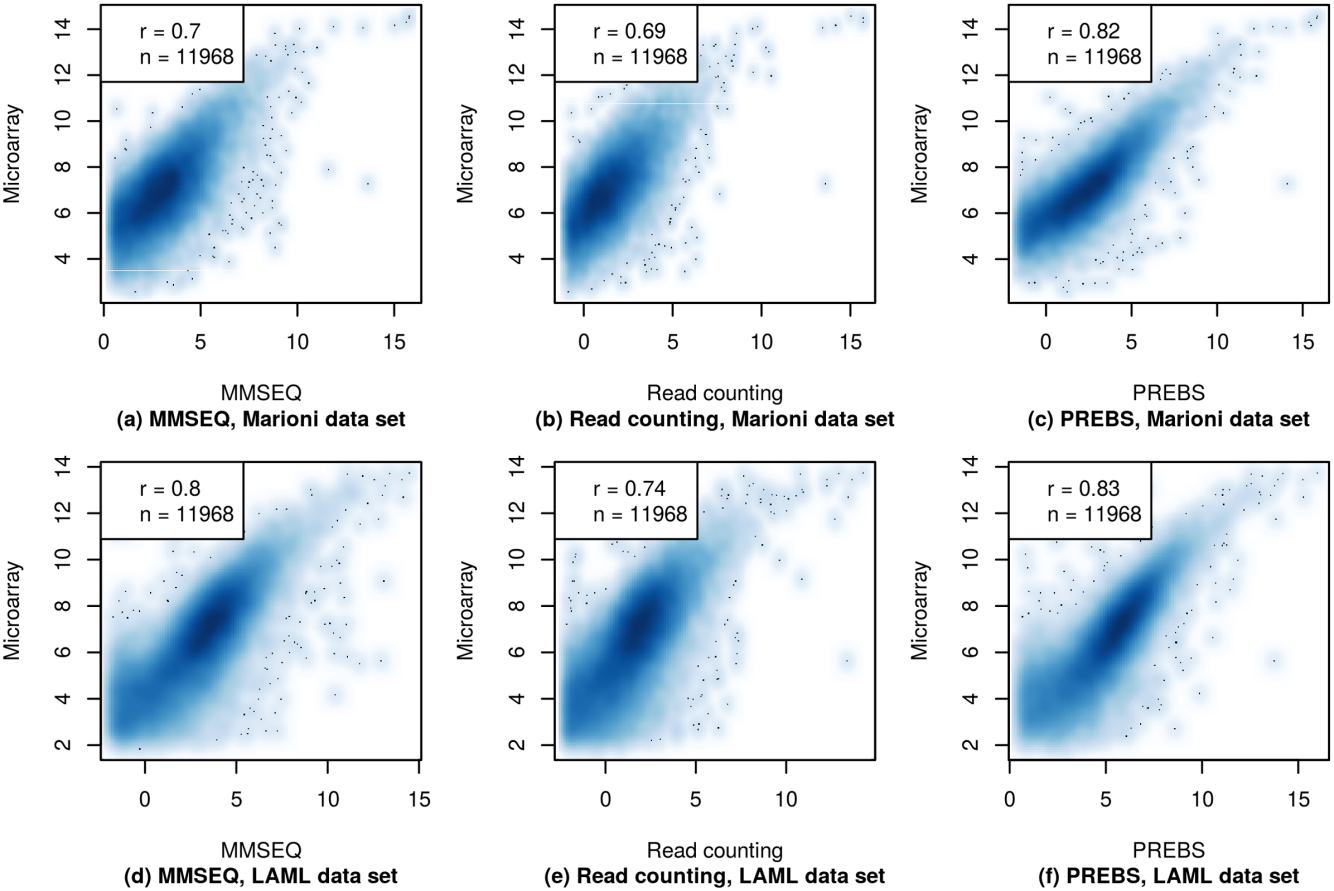
Absolute gene expression scatter plots (RPA mode). The gene expression values from three different RNA-seq data processing methods (MMSEQ, read counting and PREBS) are plotted against gene expression values from microarray. Only plots for a single sample in each data set are shown. The top row shows results for the kidney sample from the Marioni *et al.* data set and the bottom row for the 2803 sample from the LAML data set. The figures show 60% of most highly expressed genes. The legend contains Pearson correlation (*r*) and the number of genes (*n*).

To give an example of how gene expression values look like within a single sample, we provide gene expression scatter plots for the first sample in each of the data sets: kidney sample in the Marioni and 2803 sample in the LAML data set (Fig 3). The microarray gene expression estimates are plotted against sequencing-based estimates for each of the three RNA-seq data processing methods: PREBS, MMSEQ and read counting. In general, the shapes of scatter plots for all of the methods look similar, however, PREBS reaches the highest Pearson correlation both on kidney sample in the Marioni data set (*r* = 0.78) and 2803 sample in the LAML data set (*r* = 0.83).

We tested the significance of observed correlation differences for a single sample using r.test() function from psych package. The significance of difference between PREBS vs microarray correlation and read counting or MMSEQ vs microarray correlation was tested taking into account the number of genes for which the correlation is calculated. All of the observed correlation differences were significant with *p*-values lower than 10^−6^.

### Retrieval of similar microarray experiments by an RNA-seq experiment

One of our main motivations for developing the PREBS method is information retrieval, where the aim is to retrieve similar experiments based on the content, i.e. the signature of expressed genes. The higher similarity of RNA-seq and microarray data provided by PREBS processing should allow combining these two types of data more effectively. This kind of joint modelling would significantly increase the utility of methods for content-based organisation of large gene expression databases such as that of [3].

We designed an experiment to see whether the increased absolute gene expression correlation of PREBS and microarrays can be useful in a similar RNA-seq–microarray retrieval task. In this experiment we had several RNA-seq experiments with a matching microarray experiment that had to be retrieved from a database. We used the 183 microarray samples in the LAML data set, 147 of which had a matched RNA-seq pair. For each RNA-seq experiment we calculated gene expression estimate correlation with all microarray experiments. Accuracy was measured by how often the correct pair had the highest correlation. Accuracy of retrieval was calculated for all three RNA-seq data processing methods: PREBS, MMSEQ and read counting.

**Fig 4:**
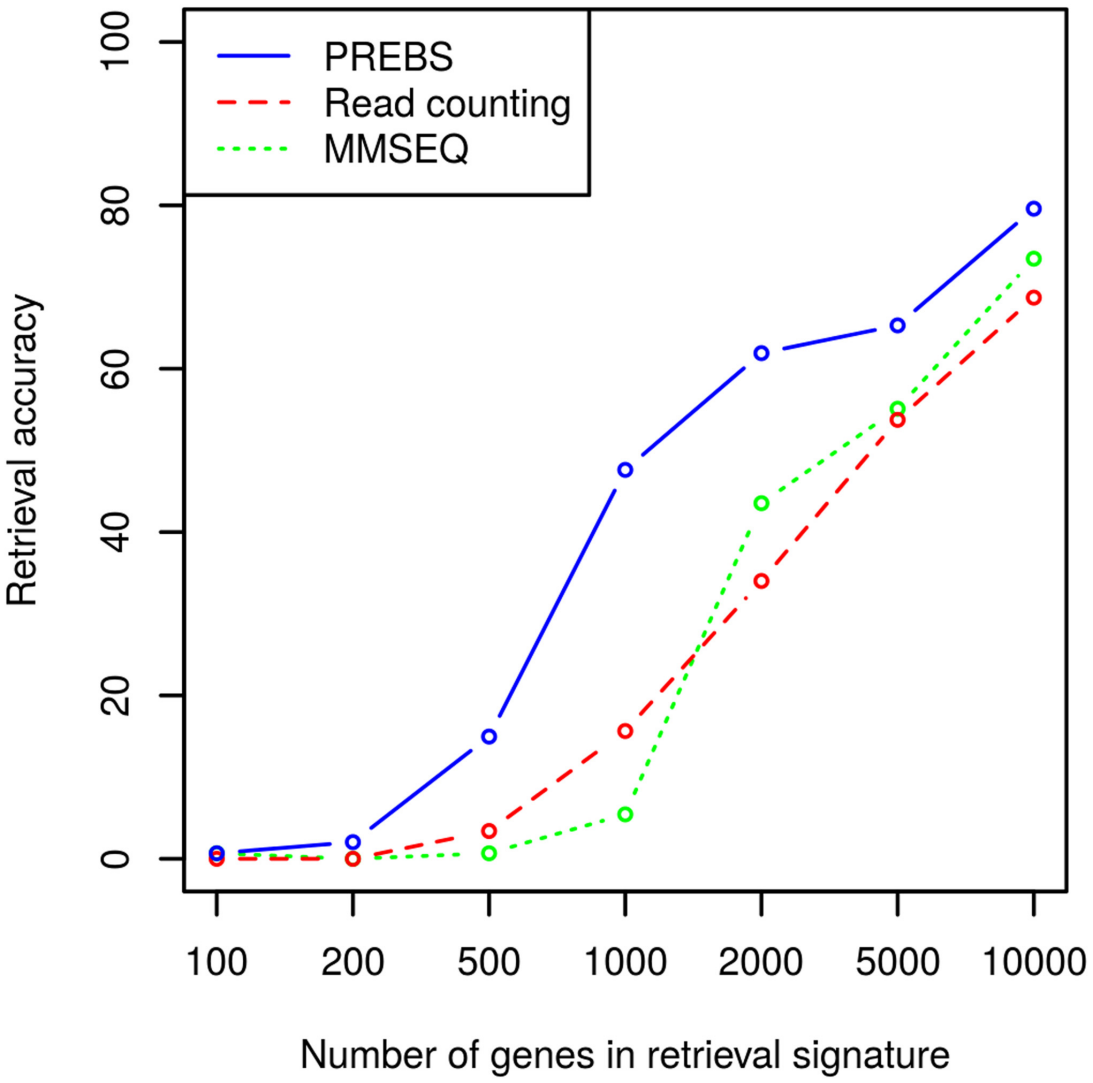
Retrieval accuracy of coupled RNA-seq–microarray experiments (RPA mode). The plot shows average precision of retrieving the corresponding microarray experiment from a large collection based on correlation with expression estimates from RNA-seq as a function of the number of genes used as the signature. Accuracy is measured as a fraction of the samples which have the largest correlation with its true pair.

To evaluate the performance of the methods using different sized signatures, we evaluated the performance of the methods with different numbers of top expressed genes. As we can see in the results in Fig 4, PREBS has clearly a better agreement with microarrays than the other RNA-seq data processing methods, especially when relatively small subsets of most highly expressed genes are used as signatures. Looking this another way, PREBS can provide similar accuracy with a signature that is significantly smaller than what is needed by the other methods, which can provide significant computational savings in modelling large databases.

### Differential expression comparison

Similarly to the absolute expression comparison, we compared the three RNA-seq data processing methods based on agreement with microarrays in differential expression measurements. For each of the methods and for microarrays we calculated log_2_ fold change values of gene expression between two states. Our comparison is limited to log_2_ fold changes instead of proper statistical differential expression testing because this would require biological replicates in RNA-seq data which are not available in the data sets used, and would be less meaningful anyway because of the different nature of the tests used on different platforms. We wish to emphasise that we do *not* recommend this procedure as a primary method of differential expression analysis for RNA-seq data. The results are reported here to better help evaluate the strengths and weaknesses of PREBS, and to suggest what is possible with cross-platform comparisons.

**Fig 5:**
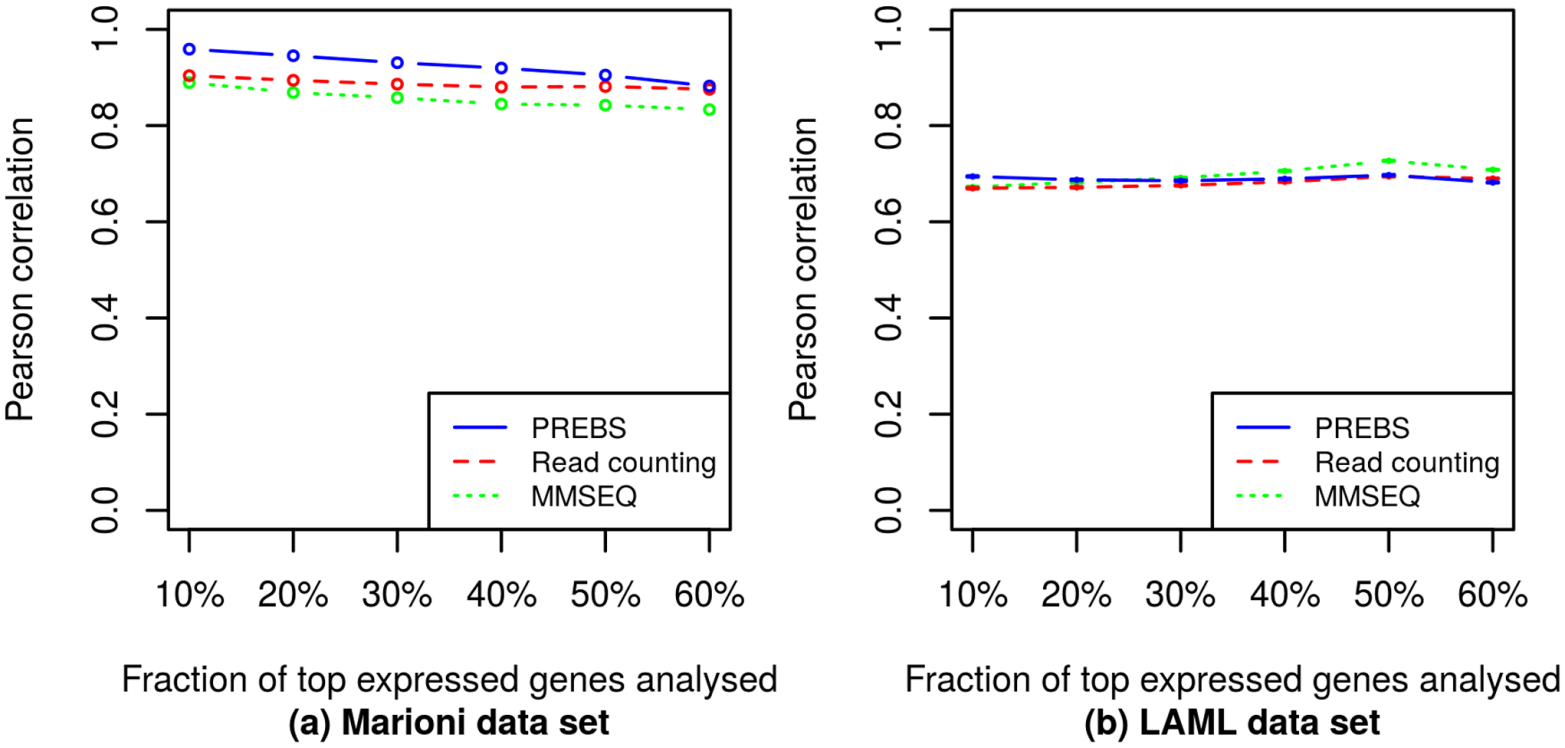
Averaged differential gene expression correlations (RPA mode). The plots show average log_2_ fold change correlations between different RNA-seq data processing methods and the microarray. Different points correspond to different numbers of top expressed genes. The correlations are averaged over all samples in the corresponding data sets: (a) the Marioni *et al.* data set, (b) the LAML data set. The error bars in LAML data set plot correspond to standard errors of the mean, although the errors are so small that top and bottom bars are merged. Error bars for Marioni data set plot could not be displayed because there is only one pair of samples for which log_2_ fold change values were calculated.

We evaluated the agreement between the three RNA-seq data processing methods and microarray by calculating Pearson correlations between sequencing-based log_2_ fold change values and microarray log_2_ fold change values. Again we did that for different fractions of top expressed genes: 10–60%. Since log_2_ fold change calculation requires two samples, we calculated them for all possible sample pairs (1 pair for the Marioni data set and (1472)=10731 pairs for the LAML data set). So in Fig 5 we provide log_2_ fold change correlations averaged over all possible sample pairs in each data set for different fractions of top expressed genes.

In contrast to the absolute expression case, we see that the differences in differential expression correlations between different methods are very small. PREBS method performs slightly better on the higher end of expression (10–20%), but slightly worse on the lower end of expression (50–60%). We can also see that the differential expression agreement is better in the Marioni data set where the expression difference between the samples is large than in the LAML data set where the samples have quite similar expression levels.

**Fig 6:**
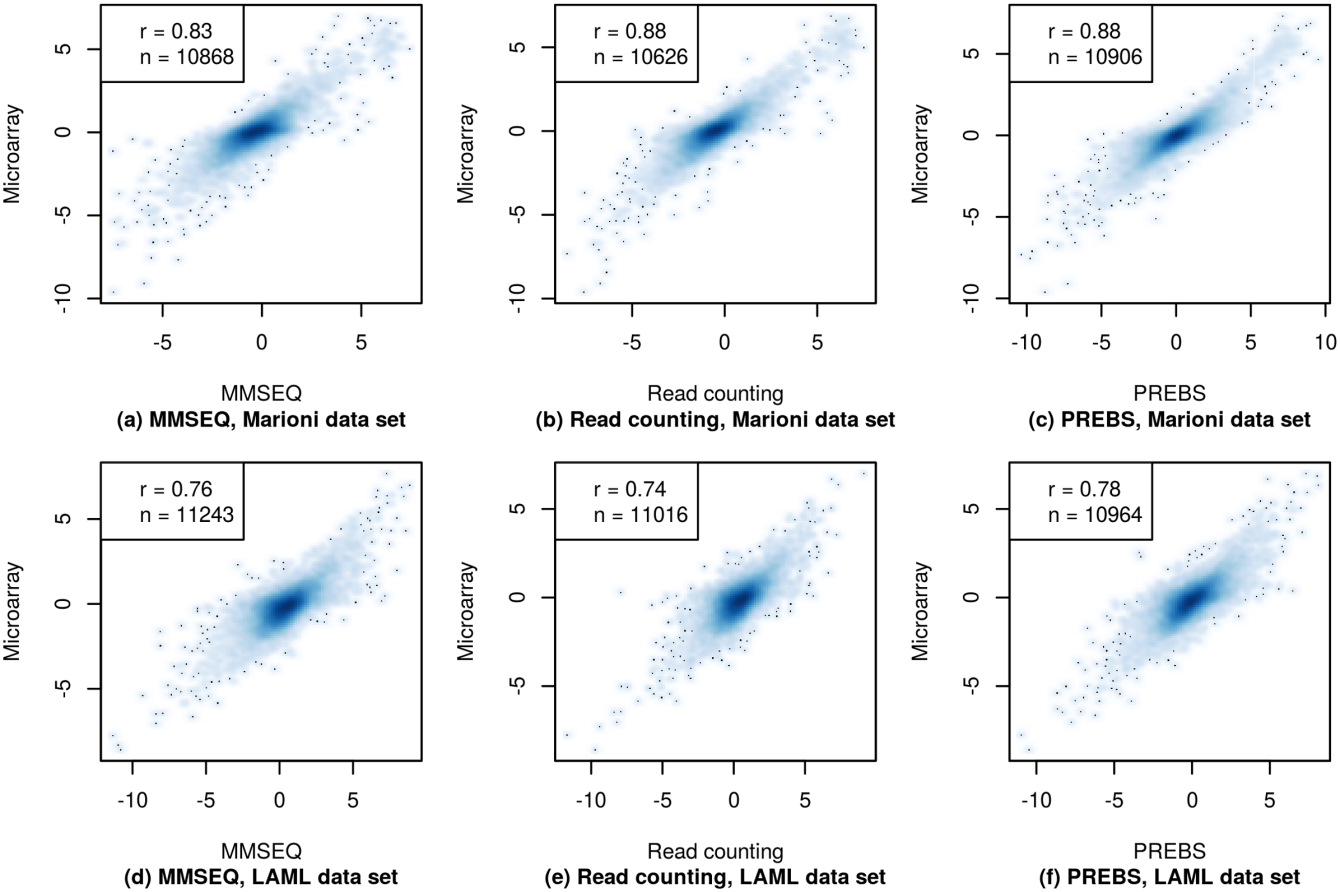
Differential expression scatter plots (RPA mode). log_2_ fold change values for differential expression estimated using different RNA-seq analysis methods plotted against corresponding microarray log_2_ fold change values. The figures show 60% of most highly expressed genes. Only plots for a single sample pair in each data set are shown. The top row shows the fold changes between the kidney and liver samples from the Marioni data set, while the bottom row shows changes between samples 2803 and 2805 from the LAML data set. The legend contains Pearson correlation (*r*) and the number of genes (*n*).

We provide an example of gene expression scatter plots for differential expression for the first pair of samples of each data set in Fig 6. Again we can see that the shapes of scatter plots look rather similar between different methods. The correlation levels differ slightly, but not as much as in absolute expression case.

**Fig 7:**
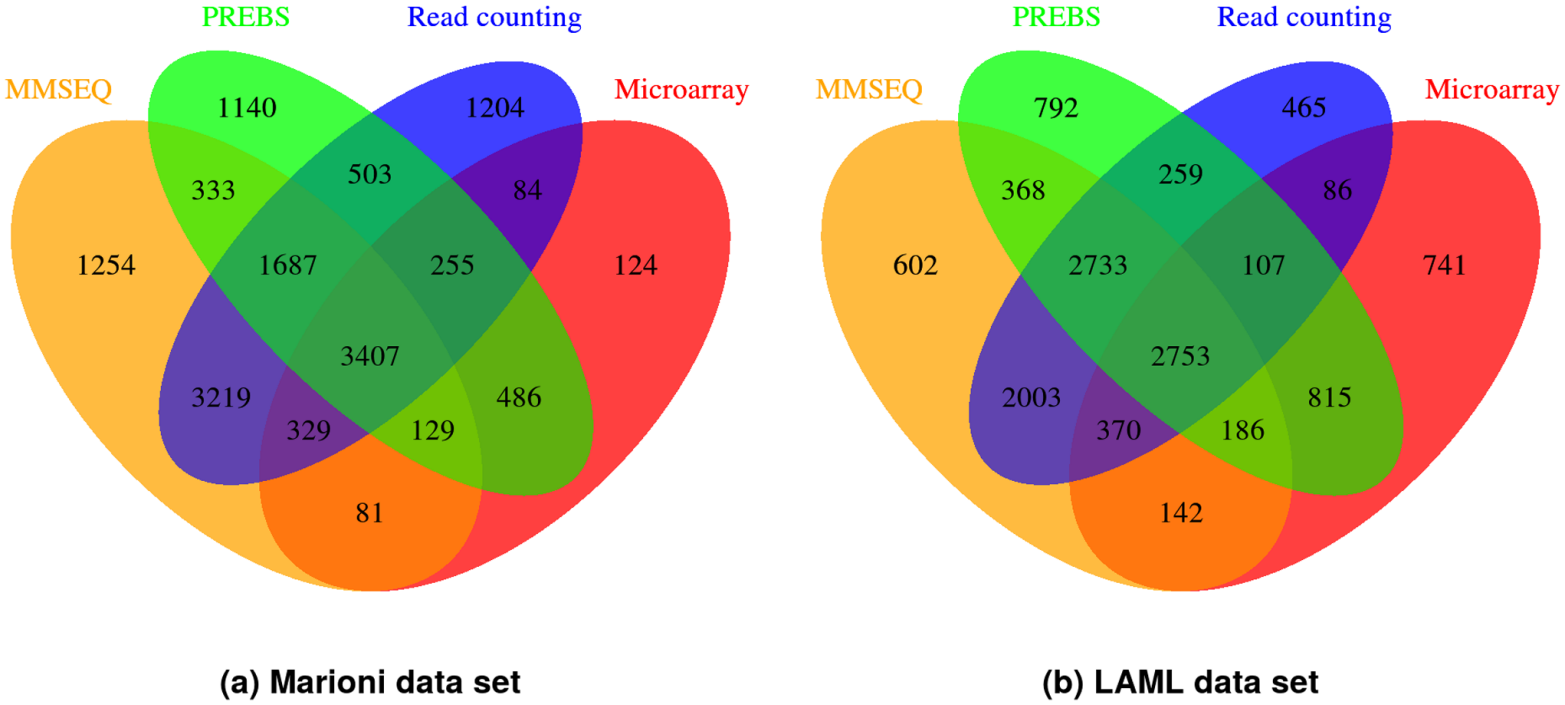
Venn diagrams of differentially expressed genes (RPA mode). The Venn diagrams illustrate the similarities of lists of genes that are called differentially expressed by different methods. We call genes with the absolute value of log_2_ fold change higher than 1.5 as significantly differentially expressed. The pairs of samples that are analysed are the same as in Fig 6 (kidney and liver for Marioni data set, 2803 and 2805 for LAML data set).

Fig 7 shows a comparison of the numbers of genes that have absolute value of log_2_ fold change greater than 1.5 (the criterion for differential expression used e.g. in [13]) for example sample pairs in both data sets. According to these results, PREBS has a better correlation with microarray results by having many more genes in common with microarrays than read counting and MMSEQ methods on both data sets. On Marioni data set PREBS has 486 differentially expressed genes that are common with microarrays while MMSEQ and read counting have only 81 and 84 respectively. On LAML data set the difference is even larger: PREBS has 815 genes common with microarrays while MMSEQ and read counting have only 142 and 86 respectively. On the other hand, both MMSEQ and read counting find a lot of differentially expressed genes that are not detected by neither PREBS nor microarray (3219 on Marioni and 2003 on LAML). The added sensitivity arises most likely because it uses read data from the whole gene regions, while PREBS restricts itself only to the gene regions where microarray probes are located. Overall, this again confirms that PREBS results agree with microarray better than MMSEQ and read counting results.

### Cross-platform differential expression

Better comparability between microarray and RNA-seq data also allows completely new operations, such as cross-platform differential expression analysis between samples measured with different technologies. This is a very difficult task because RNA-seq and microarray measures suffer from different biases, and the results of any such analysis should always be interpreted with care.

To compute the cross-platform differential expression fold change we perform an extreme quantile normalisation by replacing RNA-seq gene expression measures with microarray gene expression measures having corresponding ranks in the coupled experiment. This way, we have not changed the relative order expression levels, but made the dynamic ranges of the two platforms identical.

**Fig 8:**
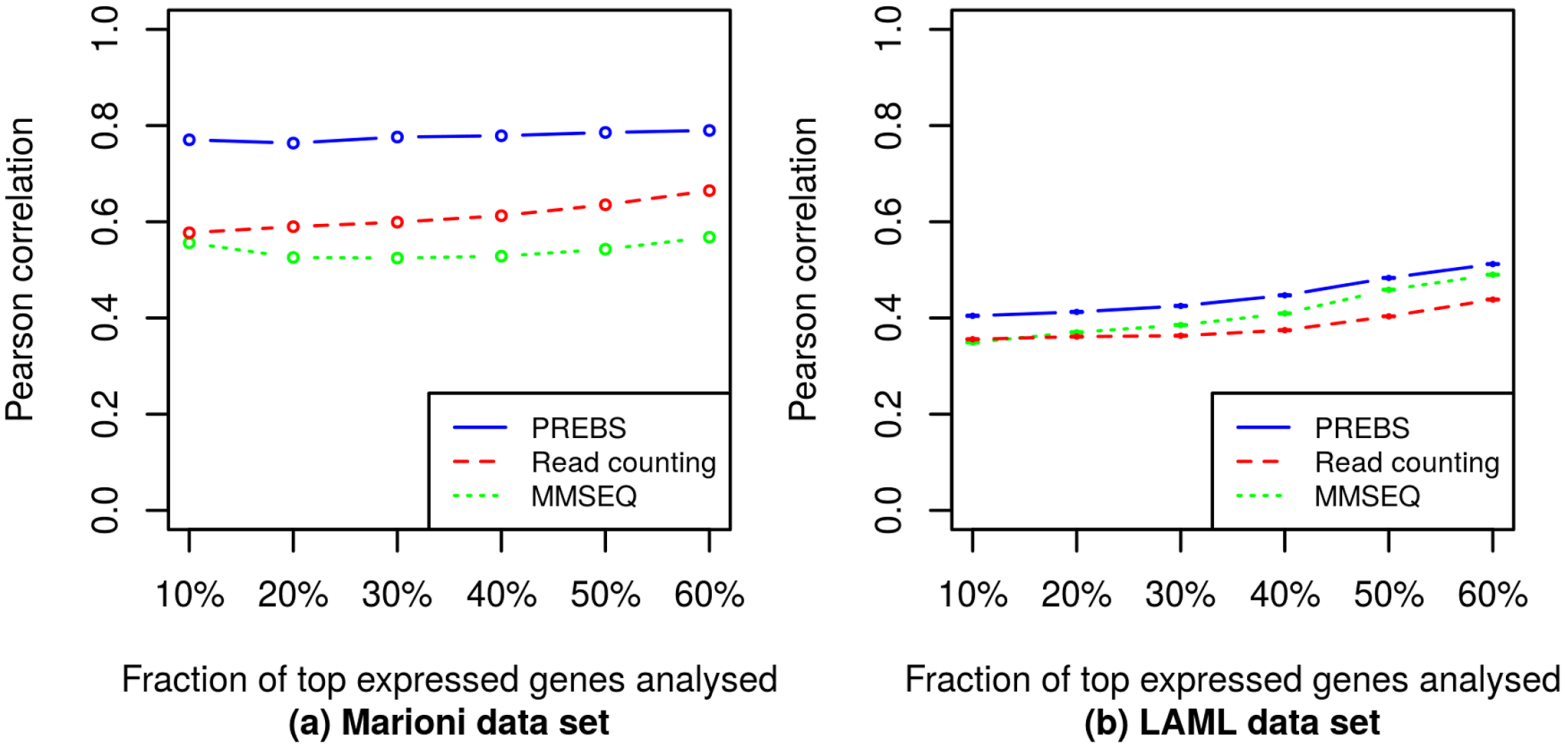
Averaged cross-platform differential gene expression correlations (RPA mode). The plots show average cross-platform differential gene expression correlations between different RNA-seq data processing methods and the microarray. Different points correspond to different numbers of top expressed genes. The correlations are averaged over all possible pairs of samples in the corresponding data sets: (a) the Marioni *et al.* data set, (b) the LAML data set.

The correlation plots of log_2_ fold changes for cross-platform differential gene expression are shown in Fig 8. We can see that PREBS has significantly better agreement with microarrays than the two other methods both on Marioni and LAML data sets and can reach a reasonable level of correlation especially with the Marioni data. The relative performances of the different methods mirror those in Fig 1 because the performance depends mainly on similarity of absolute expression measures.

### Probe set expression calculation

So far we discussed only the results where both PREBS and microarray were processed using Custom CDF files and gene expression values for Ensembl gene identifiers were acquired. However, the default way to process microarray data is using microarray probe set definitions. PREBS has an option to be run this way too, and in this way it can produce sequencing-based probe set expression values that can be directly compared with microarray probe set expression estimates.

**Fig 9:**
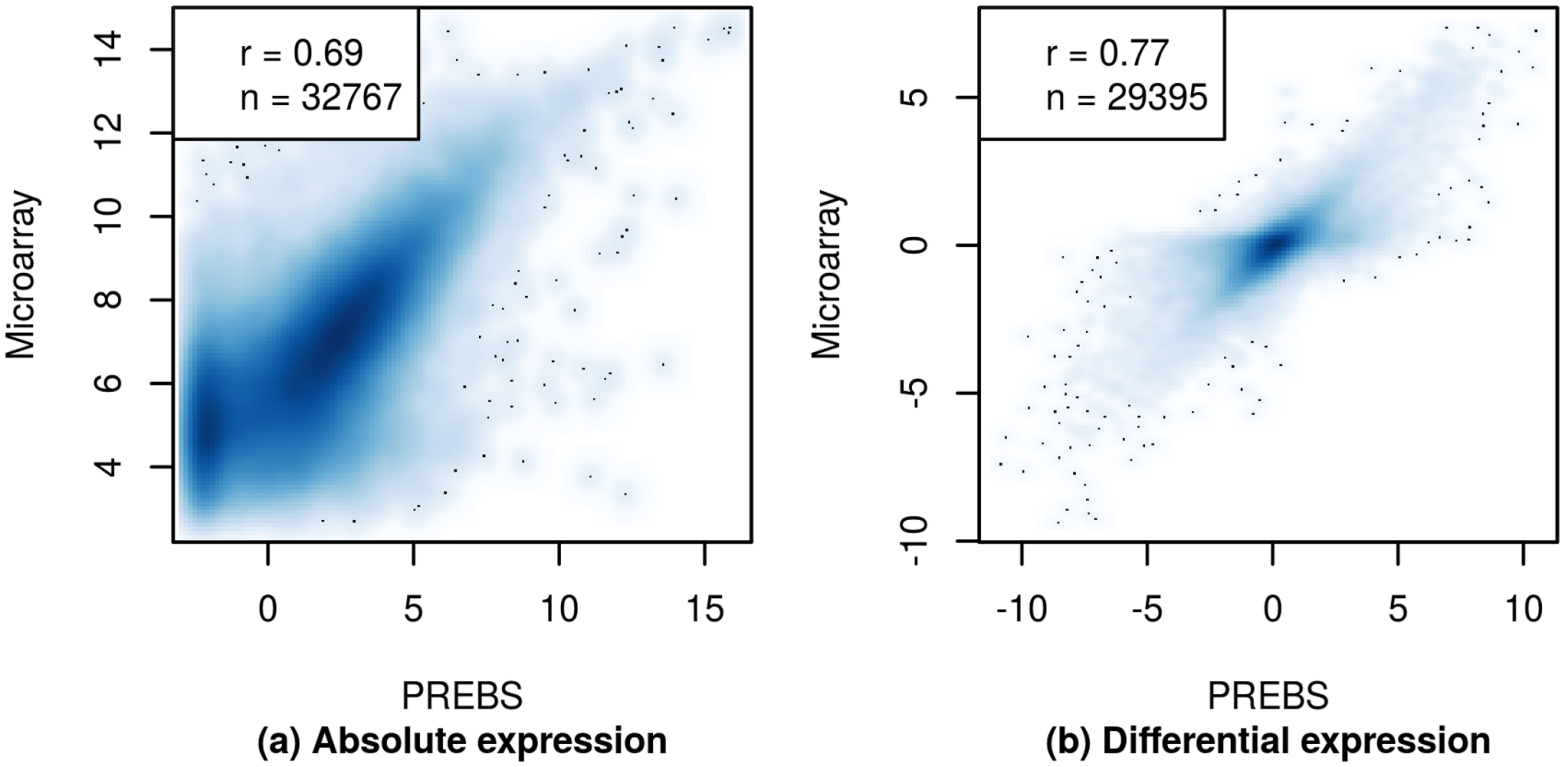
Original microarray probe set gene expression scatter plots (RPA mode). The plots show (a) estimated absolute expression values and (b) estimated log_2_ fold changes values for original microarray probe sets. The plots show 60% most highly expressed genes in the Marioni data set.

Fig 9 shows the scatter plots for absolute and differential probe set expression estimates using PREBS method on the Marioni data set. Calculating expression values for probe sets is a unique feature of PREBS and there is no easy way to do that using MMSEQ or read counting. Therefore, we did not compare PREBS with these two methods in this case.

Comparing PREBS vs microarray expression correlations of the two settings we see that the correlations for manufacturer’s probe sets (Fig 9) are slightly lower than the correlations for Ensembl genes (Figs 3 and 6). However, this is most likely due to the fact that there are many more probe sets than genes and the estimation of the corresponding individual expression levels is less reliable. Overall, PREBS provides a very reasonable level of correlation with original probe set expression levels.

## Discussion

Our results clearly demonstrate that the PREBS method is able to produce from RNA-seq data gene expression estimates that are significantly more similar to microarray estimates than standard processing pipelines. What is more, PREBS allows obtaining estimates for original microarray probe sets, which is not possible with existing methods. This will greatly aid in building integrated models of large gene expression databases that contain both microarray and RNA-sequencing data. These larger databases will help in developing more accurate machine learning methods for various predictive tasks (e.g. [33]). Efficient processing of large databases will require further work in integrating PREBS with more scalable microarray processing methods, such as [34–36].

One potential criticism against the PREBS approach is that it throws away data in the analysis. There does not however seem to be an easy way around this: microarrays only measure the expression of the probe sequences, and including RNA-seq data over other regions risks introducing confounding information due to unforeseen splicing and annotation effects. It might be possible to develop a more complex model taking all this into account, but that would be far more computationally demanding and hence less well-suited for analysis of large data collections.

PREBS greatly improves the comparability of absolute expression measures, but it does not provide a significant improvement for differential expression analysis. This may in part be explained by microarray probes that target the gene sequence suboptimally, possibly focusing only on a small fraction of its alternatively spliced isoforms. This introduces a gene-specific bias to the expression estimates. When computing the difference between multiple samples, these biases tend to cancel. The good performance of PREBS suggests that focusing on probe regions is likely a significant gene-specific bias in microarrays. Learning a model of these and other biases, such as those caused by different melting points and affinities of the probes, is an important avenue of future work, but a detailed model will require a significant amount of diverse paired RNA-seq–microarray data.

Different experimental techniques for measuring gene expression produce different results partly because they measure different things, such as different parts of the gene sequence. In this work we have presented the PREBS method which aims to eliminate this difference from RNA-seq and microarray gene expression analyses by focusing the RNA-seq summarisation to microarray probe regions. Combining this with a standard microarray data processing algorithm leads to estimates of absolute expression that are significantly more similar to ones measured from the same samples using microarrays than standard RNA-seq data processing techniques. The difference between the methods is much smaller in differential expression, presumably because gene-specific biases cancel out in the differential analysis.

Diminishing the differences between different gene expression measurement platforms paves the way for integrative modelling of large genomic data sets and big genome data applications. We have demonstrated that the PREBS approach can lead to increased accuracy in a simplified content-based genomic information retrieval task. Extending this success to a realistic integrative modelling system is a very attractive avenue of future research.

## Supporting Information

S1 Fig Averaged absolute gene expression correlations (RMA mode). The plots show average absolute gene expression correlations between different RNA-seq data processing methods and the microarray. Different points correspond to different numbers of top expressed genes. The correlations are averaged over all samples in the corresponding data sets: (a) the Marioni *et al.* data set, (b) the LAML data set. The error bars correspond to standard errors of the mean. For LAML data set the standard errors are so small that the top and bottom error bars are merged in the plot.
(TIF)

S2 Fig Absolute gene expression correlation scatter plots (RMA mode). The plots show the comparison of correlations of PREBS vs microarray and MMSEQ vs microarray for all of the samples in the LAML data set. Each point represents one sample. Two different percentages of top expressed genes are taken: (a) 10%, (b) 60%.
(TIF)

S3 Fig Absolute gene expression scatter plots (RMA mode). The gene expression values from three different RNA-seq data processing methods (MMSEQ, Read counting and PREBS) are plotted against gene expression values from microarray. Only plots for a single sample in each data set are shown. The top row shows results for the kidney sample from the Marioni *et al.* data set and the bottom row for the 2803 sample from the LAML data set. The figures show 60% of most highly expressed genes. The legend contains Pearson correlation (*r*) and the number of genes (*n*).
(TIF)

S4 Fig Retrieval accuracy of coupled RNA-seq–microarray experiments (RMA mode). The plot shows average precision of retrieving the corresponding microarray experiment from a large collection based on correlation with expression estimates from RNA-seq as a function of the number of genes used as the signature. Accuracy is measured as a fraction of the samples which have the largest correlation with its true pair.
(TIF)

S5 Fig Averaged differential gene expression correlations (RMA mode). The plots show average log_2_ fold change correlations between different RNA-seq data processing methods and the microarray. Different points correspond to different numbers of top expressed genes. The correlations are averaged over all samples in the corresponding data sets: (a) the Marioni *et al.* data set, (b) the LAML data set. The error bars in LAML data set plot correspond to standard errors of the mean, although the errors are so small that top and bottom bars are merged. Error bars for Marioni data set plot could not be displayed because there is only one pair of samples for which log_2_ fold change values were calculated.
(TIF)

S6 Fig Differential expression scatter plots (RMA mode). log_2_ fold change values for differential expression estimated using different RNA-seq analysis methods plotted against corresponding microarray log_2_ fold change values. The figures show 60% of most highly expressed genes. Only plots for a single sample pair in each data set are shown. The top row shows the fold changes between the kidney and liver samples from the Marioni data set, while the bottom row shows changes between samples 2803 and 2805 from the LAML data set. The legend contains Pearson correlation (*r*) and the number of genes (*n*).
(TIF)

S7 Fig Venn diagrams of differentially expressed genes (RMA mode). The Venn diagrams illustrate the similarities of lists of genes that are called differentially expressed by different methods. We call genes with the absolute value of log_2_ fold change higher than 1.5 as significantly differentially expressed. The pairs of samples that are analyzed are the same as in Fig 6 (kidney and liver for Marioni data set, 2803 and 2805 for LAML data set).
(TIF)

S8 Fig Averaged cross-platform differential gene expression correlations (RMA mode). The plots show average cross-platform differential gene expression correlations between different RNA-seq data processing methods and the microarray. Different points correspond to different numbers of top expressed genes. The correlations are averaged over all possible pairs of samples in the corresponding data sets: (a) the Marioni *et al.* data set, (b) the LAML data set.
(TIF)

S9 Fig Original microarray probe set gene expression scatter plots (RMA mode). The plots show (a) estimated absolute expression values and (b) estimated log_2_ fold changes values for original microarray probe sets. The plots show 60% most highly expressed genes in the Marioni data set.
(TIF)

S10 Fig Absolute expression correlation differences between RPA and RMA modes. The plots show the differences in Pearson correlation of absolute expression levels between the data processed using RPA and RMA methods in: (a) the Marioni *et al.* data set, (b) the LAML data set. Positive values mean that the RPA correlation is higher. In other words, the plot shows the difference between Fig 1 and S1 Fig.
(TIF)

S11 Fig Differential expression correlation differences between RPA and RMA modes. The plots show the differences in Pearson correlation of differential expression levels between the data processed using RPA and RMA methods in: (a) the Marioni *et al.* data set, (b) the LAML data set. Positive values mean that the RPA correlation is higher. In other words, the plot shows the difference between Fig 5 and S5 Fig.
(TIF)
