# Supplementary figures and images for: Probe Region Expression Estimation for RNA-Seq Data for Improved Microarray Comparability
Source: PLoS One. 2015 May 12;10(5):e0126545. doi: 10.1371/journal.pone.0126545 (PMC4429080; doi:10.1371/journal.pone.0126545)

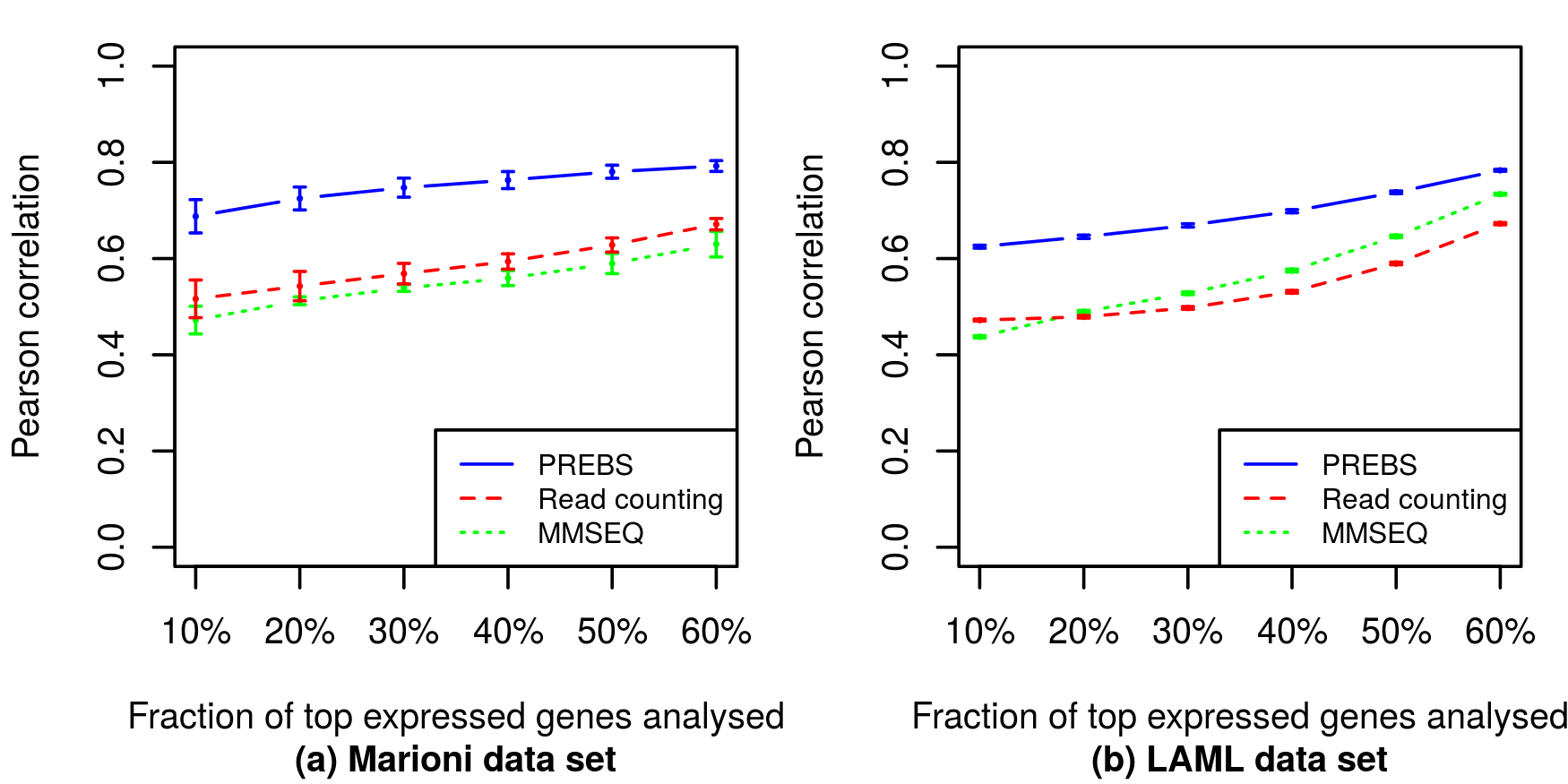

Supplement: S1 Fig — The plots show average absolute gene expression correlations between different RNA-seq data processing methods and the microarray. Different points correspond to different numbers of top expressed genes. The correlations are averaged over all samples in the corresponding data sets: (a) the Marioni et al. data set, (b) the LAML data set. The error bars correspond to standard errors of the mean. For LAML data set the standard errors are so small that the top and bottom error bars are merged in the plot. (TIF) [file pone.0126545.s001.tif]

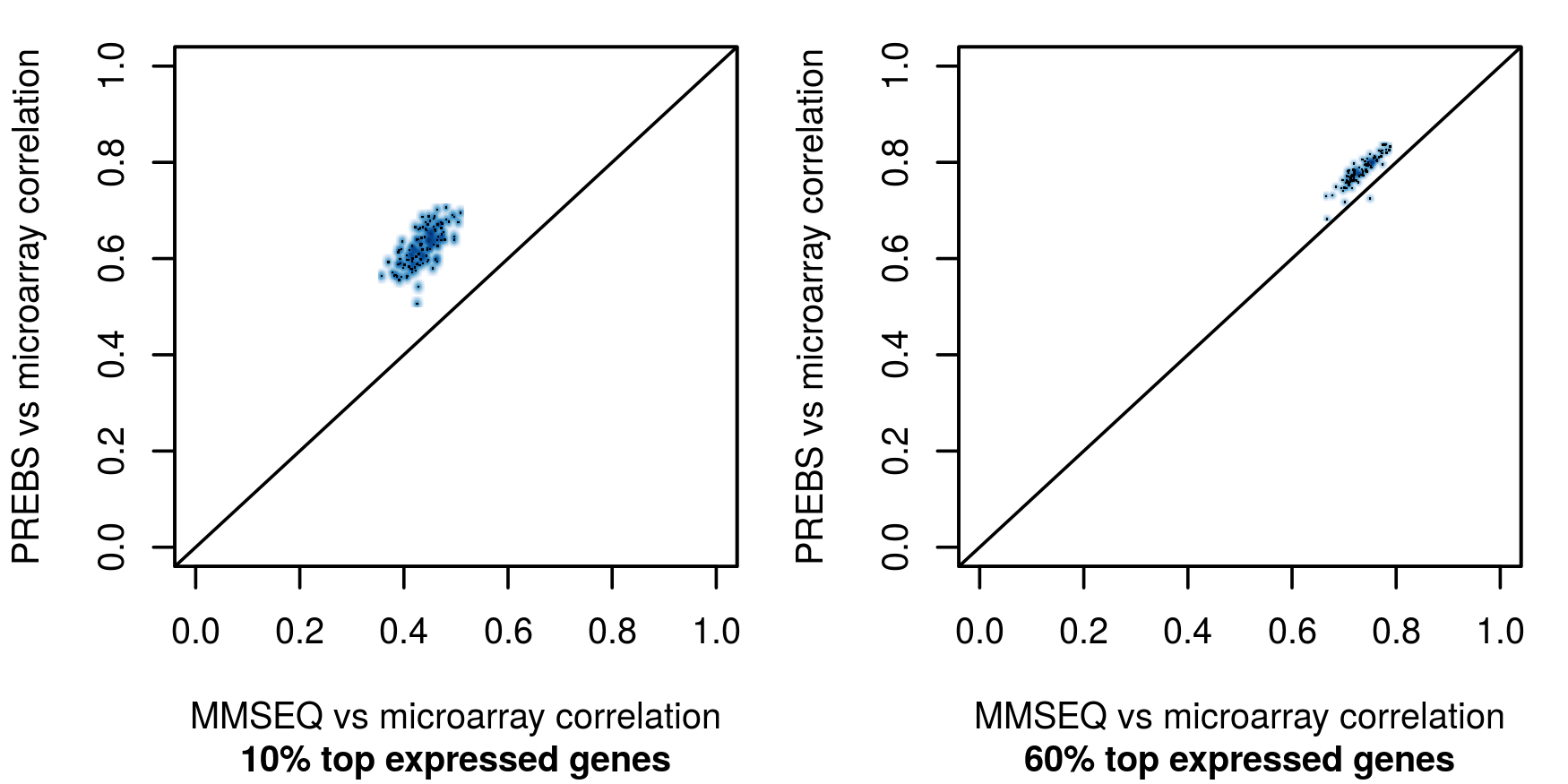

Supplement: S2 Fig — The plots show the comparison of correlations of PREBS vs microarray and MMSEQ vs microarray for all of the samples in the LAML data set. Each point represents one sample. Two different percentages of top expressed genes are taken: (a) 10%, (b) 60%. (TIF) [file pone.0126545.s002.tif]

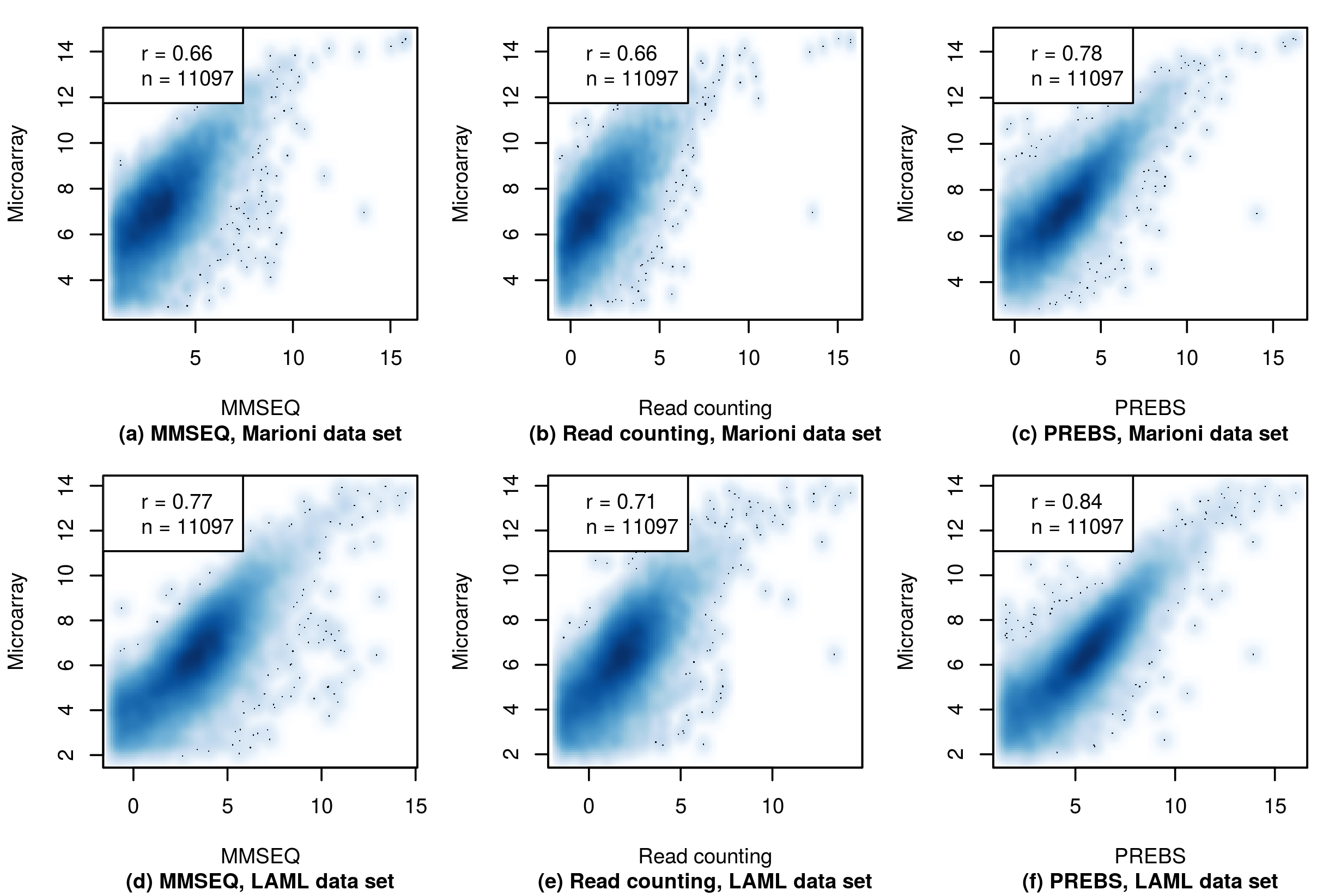

Supplement: S3 Fig — The gene expression values from three different RNA-seq data processing methods (MMSEQ, Read counting and PREBS) are plotted against gene expression values from microarray. Only plots for a single sample in each data set are shown. The top row shows results for the kidney sample from the Marioni et al. data set and the bottom row for the 2803 sample from the LAML data set. The figures show 60% of most highly expressed genes. The legend contains Pearson correlation (r) and the number of genes (n). (TIF) [file pone.0126545.s003.tif]

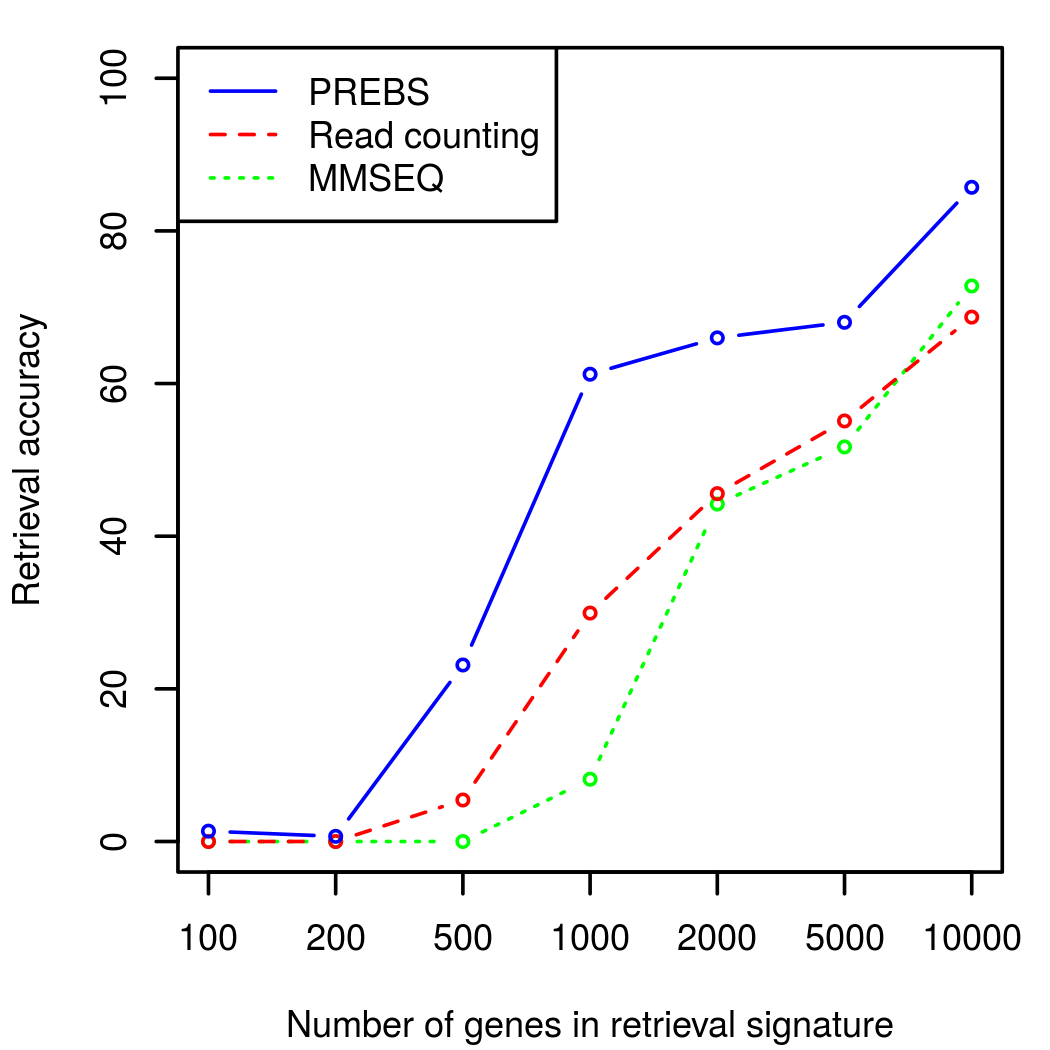

Supplement: S4 Fig — The plot shows average precision of retrieving the corresponding microarray experiment from a large collection based on correlation with expression estimates from RNA-seq as a function of the number of genes used as the signature. Accuracy is measured as a fraction of the samples which have the largest correlation with its true pair. (TIF) [file pone.0126545.s004.tif]

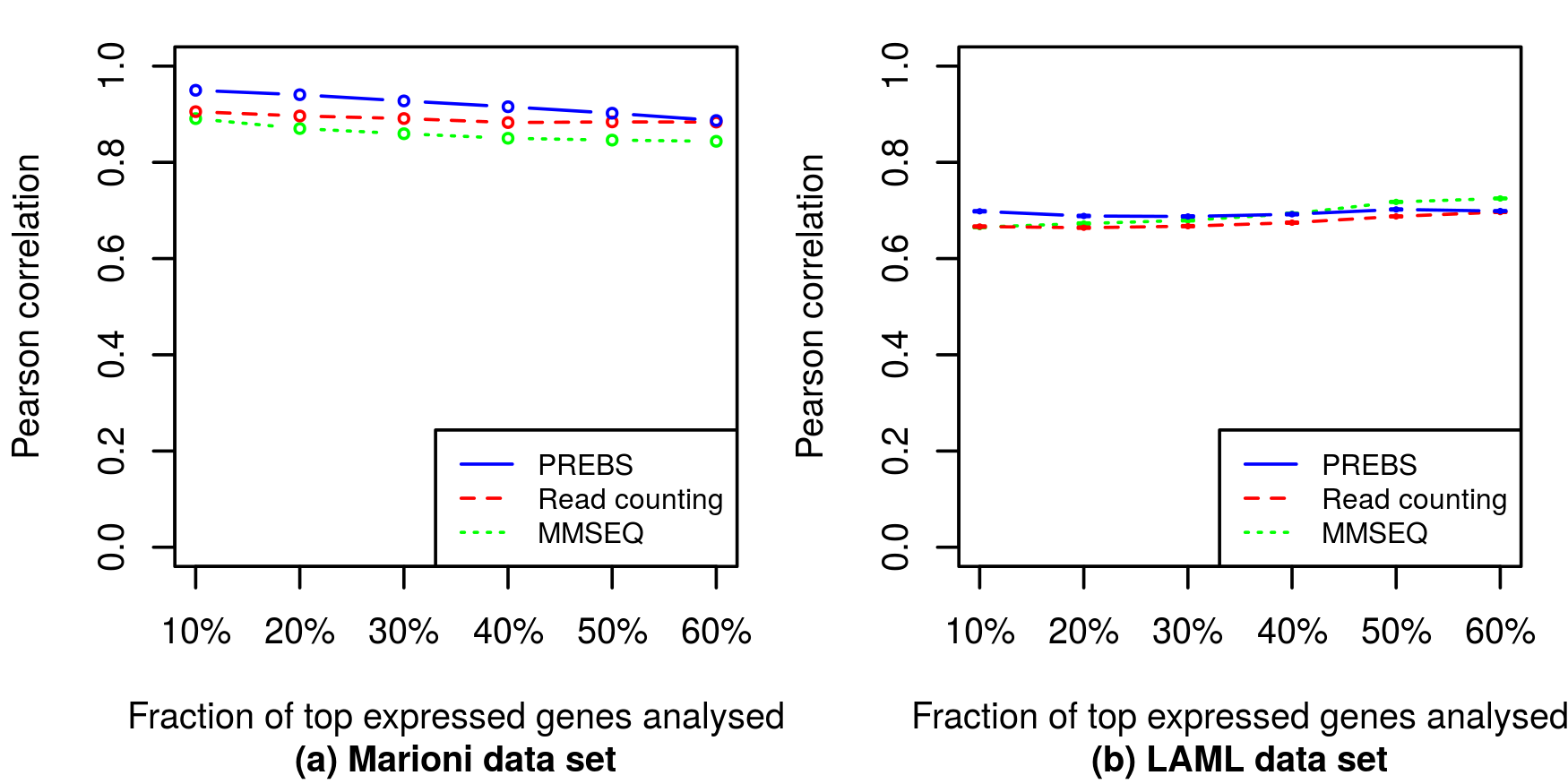

Supplement: S5 Fig — The plots show average log2 fold change correlations between different RNA-seq data processing methods and the microarray. Different points correspond to different numbers of top expressed genes. The correlations are averaged over all samples in the corresponding data sets: (a) the Marioni et al. data set, (b) the LAML data set. The error bars in LAML data set plot correspond to standard errors of the mean, although the errors are so small that top and bottom bars are merged. Error bars for Marioni data set plot could not be displayed because there is only one pair of samples for which log2 fold change values were calculated. (TIF) [file pone.0126545.s005.tif]

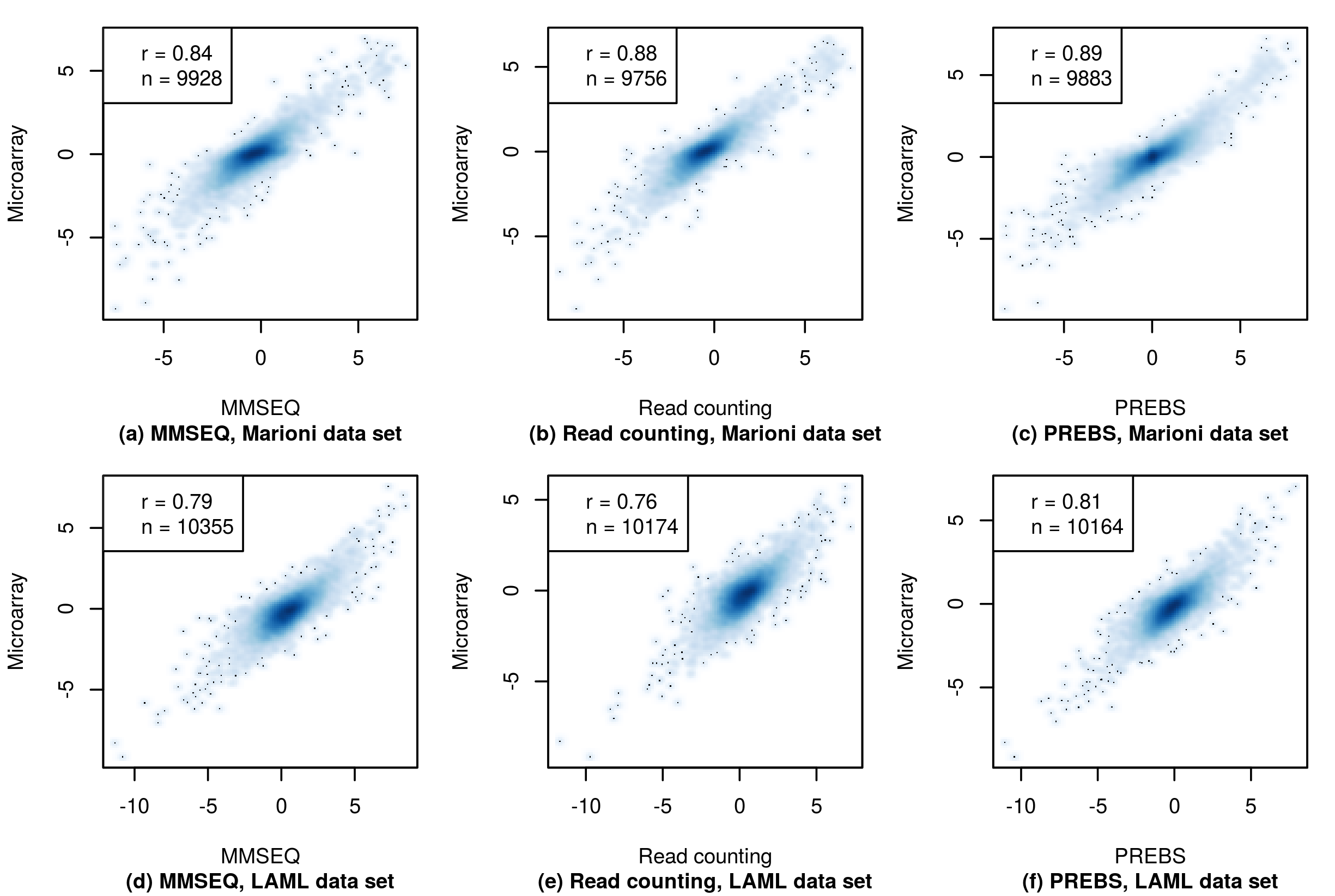

Supplement: S6 Fig — log2 fold change values for differential expression estimated using different RNA-seq analysis methods plotted against corresponding microarray log2 fold change values. The figures show 60% of most highly expressed genes. Only plots for a single sample pair in each data set are shown. The top row shows the fold changes between the kidney and liver samples from the Marioni data set, while the bottom row shows changes between samples 2803 and 2805 from the LAML data set. The legend contains Pearson correlation (r) and the number of genes (n). (TIF) [file pone.0126545.s006.tif]

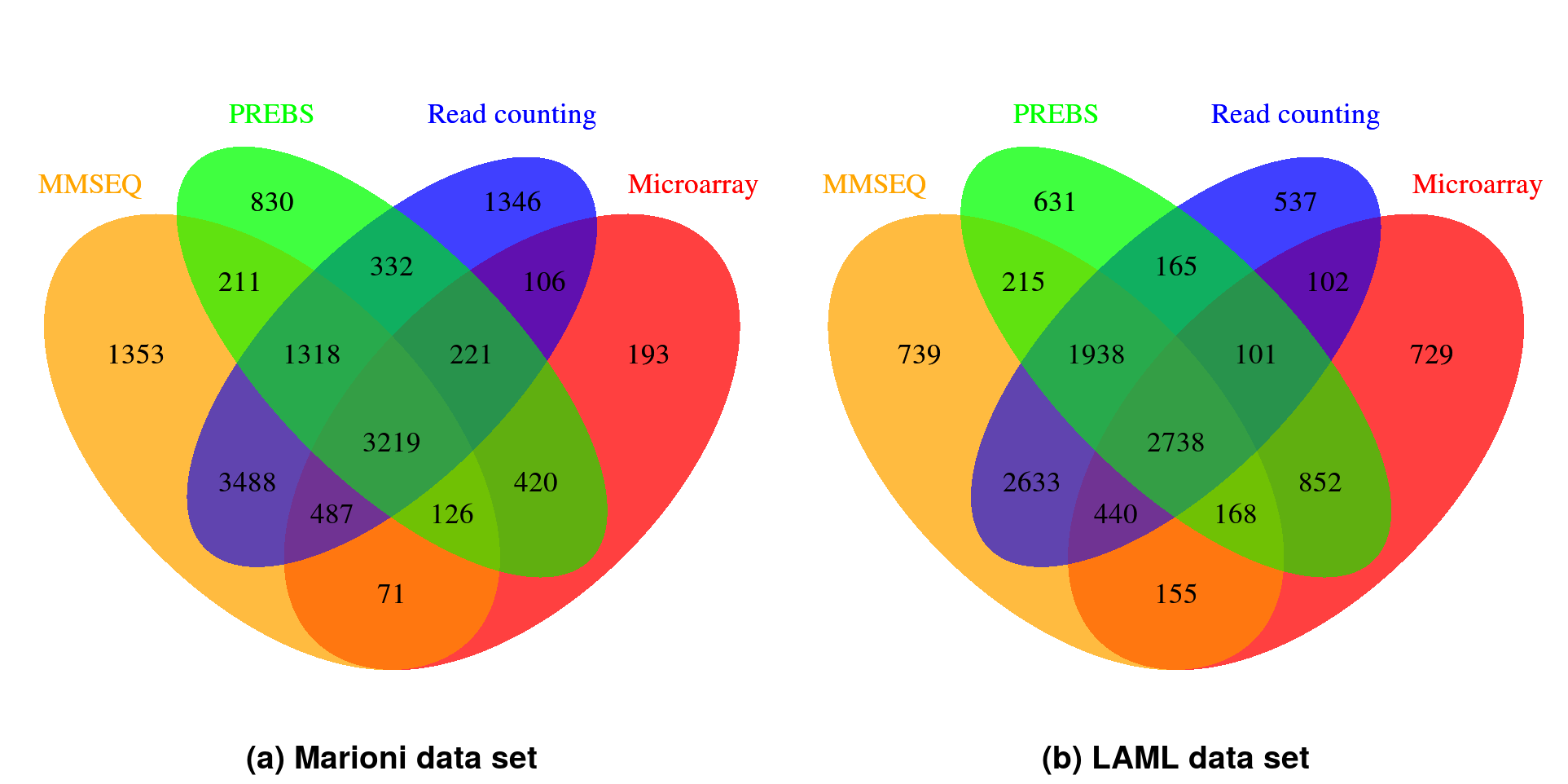

Supplement: S7 Fig — The Venn diagrams illustrate the similarities of lists of genes that are called differentially expressed by different methods. We call genes with the absolute value of log2 fold change higher than 1.5 as significantly differentially expressed. The pairs of samples that are analyzed are the same as in Fig 6 (kidney and liver for Marioni data set, 2803 and 2805 for LAML data set). (TIF) [file pone.0126545.s007.tif]

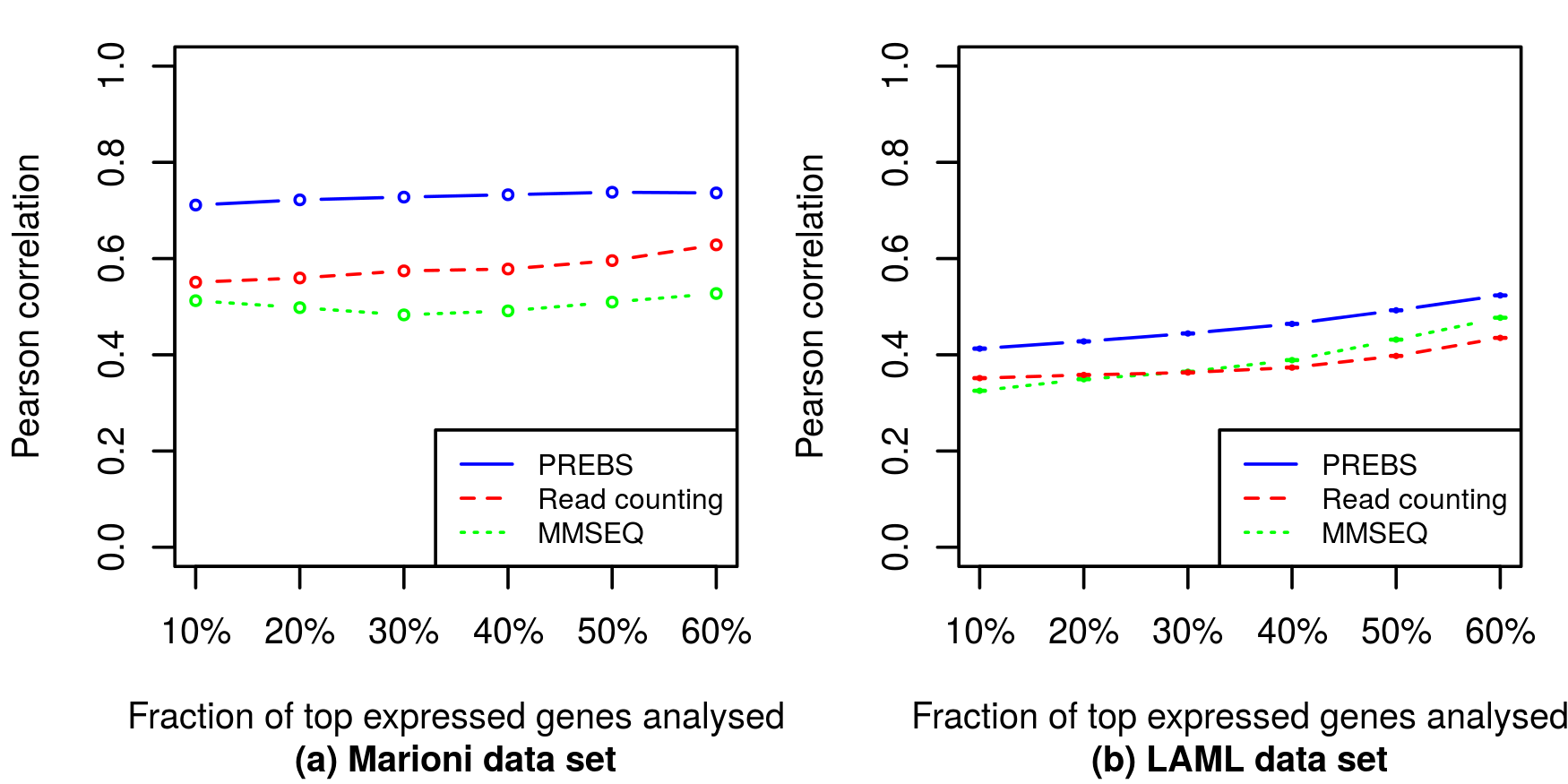

Supplement: S8 Fig — The plots show average cross-platform differential gene expression correlations between different RNA-seq data processing methods and the microarray. Different points correspond to different numbers of top expressed genes. The correlations are averaged over all possible pairs of samples in the corresponding data sets: (a) the Marioni et al. data set, (b) the LAML data set. (TIF) [file pone.0126545.s008.tif]

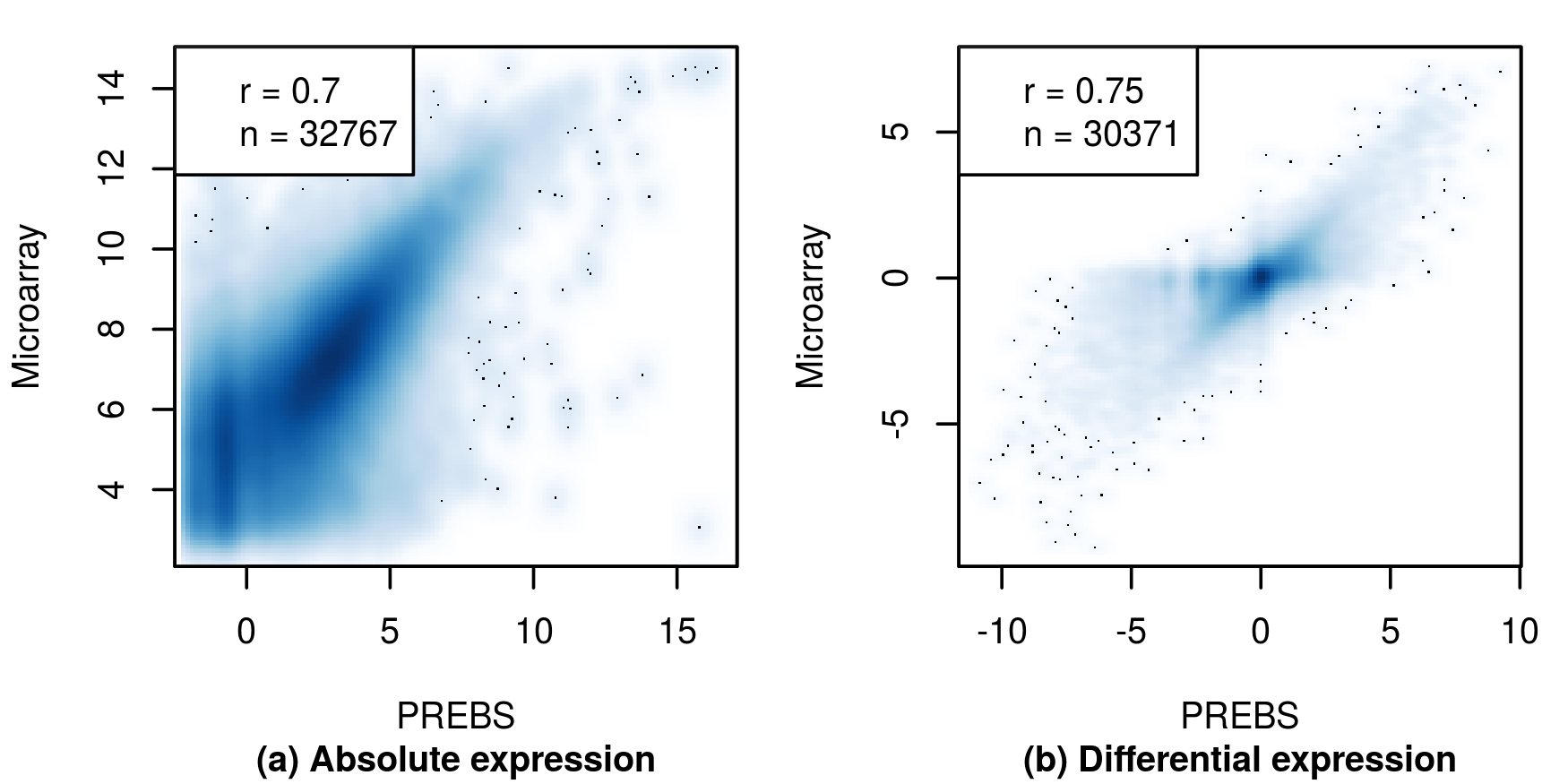

Supplement: S9 Fig — The plots show (a) estimated absolute expression values and (b) estimated log2 fold changes values for original microarray probe sets. The plots show 60% most highly expressed genes in the Marioni data set. (TIF) [file pone.0126545.s009.tif]

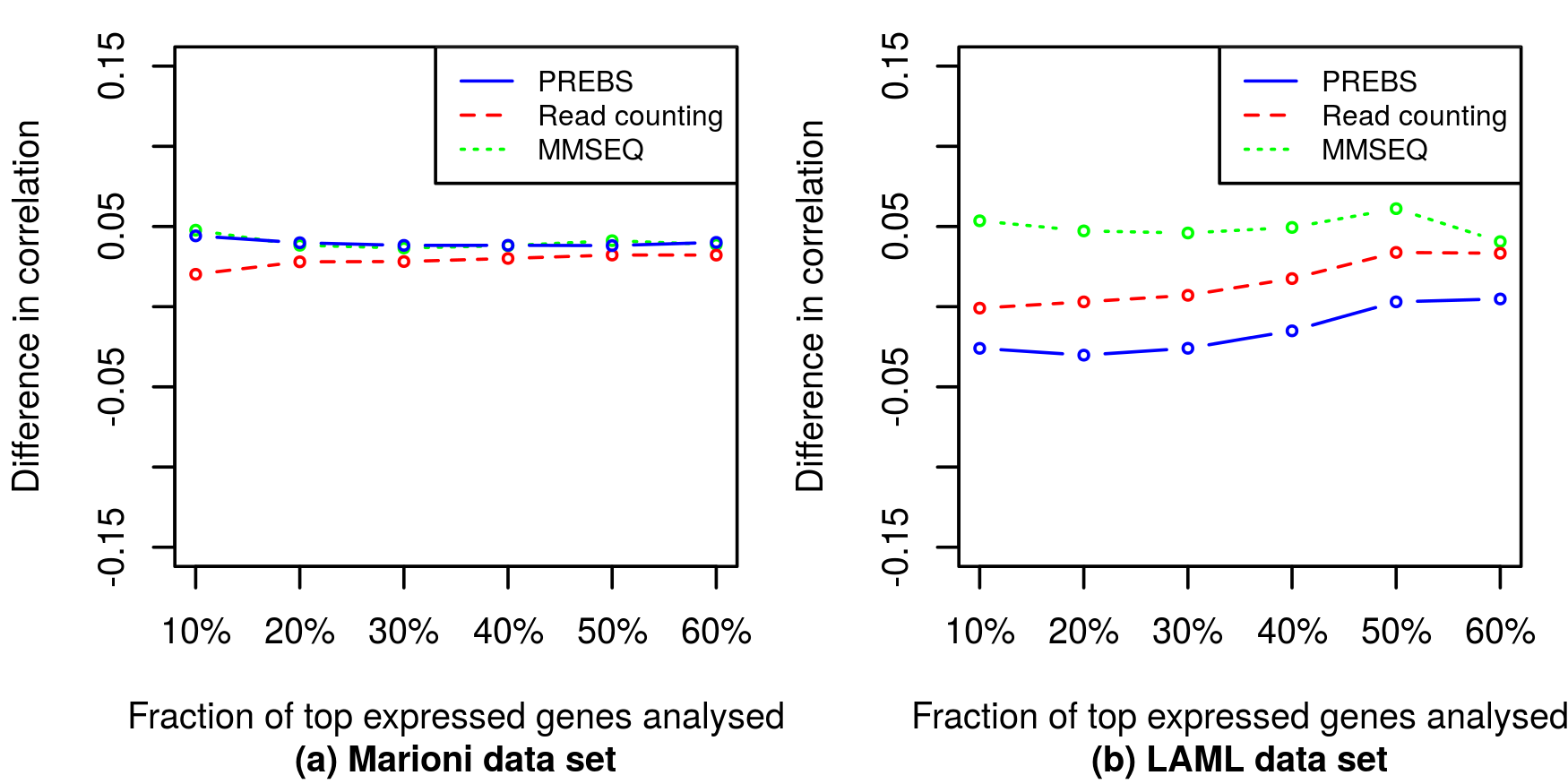

Supplement: S10 Fig — The plots show the differences in Pearson correlation of absolute expression levels between the data processed using RPA and RMA methods in: (a) the Marioni et al. data set, (b) the LAML data set. Positive values mean that the RPA correlation is higher. In other words, the plot shows the difference between Fig 1 and S1 Fig. (TIF) [file pone.0126545.s010.tif]

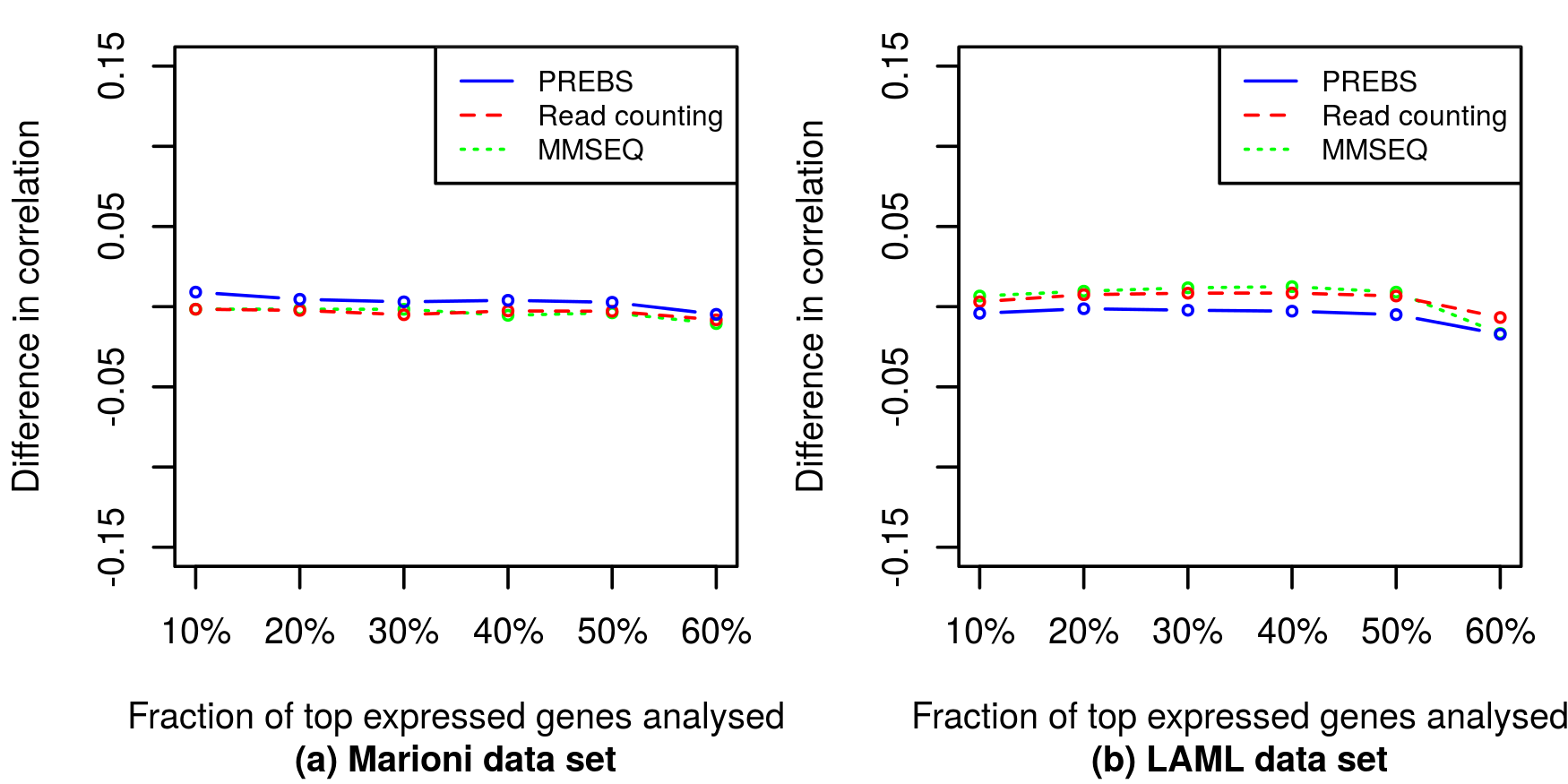

Supplement: S11 Fig — The plots show the differences in Pearson correlation of differential expression levels between the data processed using RPA and RMA methods in: (a) the Marioni et al. data set, (b) the LAML data set. Positive values mean that the RPA correlation is higher. In other words, the plot shows the difference between Fig 5 and S5 Fig. (TIF) [file pone.0126545.s011.tif]
